# Generalized metabolic flux analysis framework provides mechanism-based predictions of ophthalmic complications in type 2 diabetes patients

**DOI:** 10.1007/s13755-023-00218-x

**Published:** 2023-03-29

**Authors:** Arsen Batagov, Rinkoo Dalan, Andrew Wu, Wenbin Lai, Colin S. Tan, Frank Eisenhaber

**Affiliations:** 1 Mesh Bio Pte. Ltd., 10 Anson Rd, #22-02, 079903 Singapore, Singapore; 2 Department of Endocrinology, Tan Tock Seng Hospital, Singapore, Singapore; 3 Lee Kong Chian School of Medicine, Nanyang Technological University, Singapore, Singapore; 4 Fundus Image Reading Center, National Healthcare Group Eye Institute, Singapore, Singapore; 5 Tan Tock Seng Hospital, National Healthcare Group Eye Institute, Singapore, Singapore; 6 Duke-NUS Medical School, Singapore, Singapore; 7 Bioinformatics Institute (BII), Agency for Science, Technology and Research (A*STAR), Singapore, Singapore; 8 Genome Institute of Singapore (GIS), Agency for Science, Technology and Research (A*STAR), Singapore, Singapore; 9 School of Biological Science (SBS), Nanyang Technological University, Singapore, Singapore

**Keywords:** Metabolic flux analysis, Digital twin, Diabetes, Diabetic complications, Retinopathy, Cataract

## Abstract

Chronic metabolic diseases arise from changes in metabolic fluxes through biomolecular pathways and gene networks accumulated over the lifetime of an individual. While clinical and biochemical profiles present just real-time snapshots of the patients’ health, efficient computation models of the pathological disturbance of biomolecular processes are required to achieve individualized mechanistic insights into disease progression. Here, we describe the Generalized metabolic flux analysis (GMFA) for addressing this gap. Suitably grouping individual metabolites/fluxes into pools simplifies the analysis of the resulting more coarse-grain network. We also map non-metabolic clinical modalities onto the network with additional edges. Instead of using the time coordinate, the system status (metabolite concentrations and fluxes) is quantified as function of a generalized extent variable (a coordinate in the space of generalized metabolites) that represents the system’s coordinate along its evolution path and evaluates the degree of change between any two states on that path. We applied GMFA to analyze Type 2 Diabetes Mellitus (T2DM) patients from two cohorts: EVAS (289 patients from Singapore) and NHANES (517) from the USA. Personalized systems biology models (digital twins) were constructed. We deduced disease dynamics from the individually parameterized metabolic network and predicted the evolution path of the metabolic health state. For each patient, we obtained an individual description of disease dynamics and predict an evolution path of the metabolic health state. Our predictive models achieve an ROC-AUC in the range 0.79–0.95 (sensitivity 80–92%, specificity 62–94%) in identifying phenotypes at the baseline and predicting future development of diabetic retinopathy and cataract progression among T2DM patients within 3 years from the baseline. The GMFA method is a step towards realizing the ultimate goal to develop practical predictive computational models for diagnostics based on systems biology. This tool has potential use in chronic disease management in medical practice.

**Supplementary Information:**

The online version contains supplementary material available at 10.1007/s13755-023-00218-x.

## Introduction

In the current understanding, a metabolic disorder can be traced to a particular set of biochemical reactions and metabolites, whose abnormal changes lead to syndrome manifestation and progression. These altered metabolic states typically have complex causes and develop due to individual genetic variations, changes in the environment, behavioral factors, lifestyle, or as a side effect of disease treatment [1, 2]. As these alterations are associated with chronic disease and aging, understanding the trend of their long-term development is vital.

Despite advances in large-scale and high-precision techniques for metabolite measurements and their successful applications in identification of certain inborn metabolic disorders, quantifying the progress of chronic diseases remains challenging [3–5]. To enable broad adoption of large-scale metabolic analysis in the clinical setting, we need to bridge several major gaps. First, the high-throughput analytical techniques are frequently not as robustly established as clinical assays, which include reference ranges, standards of reproducibility, and validated metrics of sensitivity and specificity [4, 6–8]. Second, the lack of robust and flexible bioinformatics frameworks and computational approaches hinders contextualizing metabolic models with rich clinical meta-data and physiological readings [7, 9–11]. Third, the issue of time scale is pertinent in a clinical application with processes equilibrating within hours and days, whilst subtle drifts along health state trajectories could occur in the time frame of years and decades.

Mathematical methods developed within the metabolic modeling research area, such as enzymatic kinetic models, metabolic flux analysis, or stochastic models, were proposed to analyze metabolic systems. Traditional enzymatic kinetics models using the Michaelis–Menten and the Briggs–Haldane equations [12], have met a great success in describing the dynamics of isolated biochemical reactions in vitro. Since these models essentially describe dynamics of intermediate states of any bound substrate, they are general enough to also describe elementary behavior of more complex systems, ranging from bacterial growth [13] to physiological processes [14–16] and epidemiology [17, 18]. The following three examples of kinetic models application with increasing complexity (an enzyme study, an in vivo metabolite dynamics, a particle absorption study) illustrate opportunities and limitations of such an approach.

- Standard enzymatic kinetics analysis is applied to quantify the effects of several genetic variants of the arachidonate 15-lipoxygenase type II, an enzyme with a potential role in the development of coronary artery disease [16]. The authors determined mutation-specific kinetic parameters. However, the link between the kinetic model and the process of atherosclerotic plaque formation remains opaque.
- In a clinical study of glucose disposal with 88 healthy volunteers receiving four types of insulin infusions, kinetic parameters of glucose concentration for an M–M model of glucose metabolism were determined [14]. The authors found the kinetic constants insulin concentration-dependent, probably as a result of their model representing the entire metabolic mechanism only in parts.
- Study [15] applied the M–M-like model to describe pulmonary absorption kinetics of particles in the lungs of rats. The authors found their generalized kinetic model correctly reproducing the clearance rate of all four studied dust types at the phenomenological level but without a biomolecular mechanistic explanation.

At the same time, kinetic models have been proven of limited value for clinical relevant applications, because the models require a large number of biochemical reaction parameters, the physiological values of which are difficult to determine in a clinical setting.

In contrast, MFA allows a more coarse-grain representation of a complex biochemical system of reactions and binding processes as a composition of metabolic fluxes connected with stoichiometric and mass balance relations [19–23]. This methodology has been applied to prokaryotic and eukaryotic organisms on the level from individual pathways [19, 21, 23] to whole genome scale [24]. Most importantly, unlike enzymatic kinetic models, MFA explicitly relates the dynamics of metabolites to complex phenotypes [21, 25, 26]. This methodology enables the discovery of control mechanisms emerging on the level of entire biochemical pathways and networks [20, 21, 27].

Typically, MFA computational models in clinical application studies include the basics of the carbon metabolism network (pyruvate metabolism, tri-carbon cycle, glyoxylate shunt, etc.) complemented with selected pathways from the amino acid, lipid or other types metabolic or signaling network elements if necessary. With detail up to individual reactions, the models are just focused of specific aspects of the networks behavior and do not look into potentially more global metabolism changes. The limited opportunities provided by such type of modelling are illustrated by the study from Gregory et al. [28] who explored the impact of various FTL3 inhibitors on glutamine utilization in patients with acute myeloid leukemia. Yet, the model was sufficient to assess flux changes caused by combined inhibitor application and to delineate glutathione depletion as major mechanism of leukemic cell elimination.

The work of Karlstädt et al. [29] is an example where multiple state-of-the-art methods of computational modeling complement each other to address the complexity of the studied system. The authors analyzed cardiomyocyte glycolysis kinetics to reveal and elucidate the underlying control mechanisms at the metabolic and protein levels and experimentally validated predictions made in their in silico studies. They applied MFA to obtain the steady-state flux distribution, which maximized ATP production in the muscle tissue [23]. The MFA analysis allowed them to quantify systems-wide contributions of individual reaction rates and perturbations in individual metabolic parameters. To overcome the inherent limitation of MFA not addressing changes in metabolite concentrations, the simulated system was expanded with M–M-like equations. This allowed the authors to simulate metabolite concentrations and flux rates as a function of time and to predict steady-state fluxes and metabolite concentrations [29].

The human health state, being a specific case of a phenotype at a given point of a disease development trajectory, can thus be analyzed using MFA in principle. Yet, at present, the formalism is not ready for this purpose. There are several issues. First, the number of parameters in MFA models grows linearly with the number of reactions. Although this makes MFA a more scalable approach to build mechanistic models of complex systems, the number of parameters in biochemical reaction-oriented, detailed models is still enormous and finding proper values for them is problematic. Second, the metabolite concentrations typically used in MFA calculations are not commonly available from clinical laboratories. Third, at the same time, many biochemical and physiological parameters, such as urine albumin or blood pressure, currently measured in clinics, are not incorporated into MFA models.

The GMFA approach introduced in this work implements a number of methodical innovations that enhance the suitability of MFA for clinical applications.

1. To reduce the demand on the input data scale, we apply GMFA to a reduced, coarse-grain metabolic network, wherein individual metabolic entities/fluxes are grouped in accordance with biological processes.
2. We map non-metabolic clinical modalities and measurements onto the metabolic network.
3. To make health states and their progression comparable across patients, we replace the time variable with the extent variable quantifying the degree of progression between two extreme health states—the healthy state and the fully manifested disease state, assuming quasi-linearity of metabolite concentration changes along the trajectory of states.

We applied our methodology to the analysis of observational data of two cross-sectional cohorts of diabetes type 2 patients. We constructed a simplified metabolic map including key components of glucose and lipid metabolism, onto which physiological characteristics, for example, the pulse wave velocity and carotid intima-media thickness were mapped. With statistical rigor, we show that fluxes calculated with the data from patients are predictive for the development of individual ophthalmic complications in type 2 diabetes.

## Methods

### Study design and population data

To evaluate the applicability of the proposed methods to clinical data, we studied the EVAS multi-ethnic cohort of 289 patients with type 2 diabetes visiting a tertiary medical center in Singapore [30] (data collected in 2015–2020) and the NHANES multi-ethnic multi-centre general population cohort obtained in the National Health and Nutrition Examination Survey carried out in the United States (data collected in 1999–2018) [31]. From the pool of 6652 available NHANES patients with type 2 diabetes history, we selected only 517, whose measured data modalities sufficiently overlap with those of EVAS (see Table 1 for the list of respective NHANES variables).

**Table 1:** Baseline clinical and biochemical characteristics of the observational cohort study

EVAS Variable	NHANES variable	EVAS summary	NHANES summary
N total	–	289	517
Males (%)	riagender	144 (50)	257 (50)
Ethnicity: n (%)			
Chinese	–	174 (60)	NA
Malays	–	48 (17)	NA
Indians	–	67 (23)	NA
Other	ridreth1	0	517 (100)
Age years, Mean (SD)	ridageyr	54.3 (11.14)	60 (14.68)
T2DM Duration, years, Median (IQR)	did040	11 (5-17)	11 (5-18)
Hypertension duration years, Median (IQR)	NA	6.5 (0-13)	NA
Hyperlipidemia duration years, Median (IQR)	NA	7 (2-13)	NA
BMI kg/cm^2^, Mean (SD)	bmxbmi	27.7 (4.99)	32.2 (7.33)
Systolic BP, mm Hg, Mean (SD)	bpxsy	133.2 (14.78)	129.6 (20.12)
Fasting glucose mmol/L, mean (SD)	lbxglu, lbxglusi	8.89 (3.18)	9.14 (3.80)
HbA1c %, mean (SD)	diq280	8.60 (1.84)	7.47 (2.39)
Total Cholesterol mmol/L, Mean (SD)	lbxtc, lbxtcsi	4.39 (1.09)	4.53 (1.09)
HDL-Cholesterol mmol/L, Mean (SD)	lbdhdd, lbdhddsi	1.12 (0.30)	1.29 (0.36)
LDL-Cholesterol mmol/L, Mean (SD)	lbdldl, lbdldlsi	2.51 (0.84)	2.53 (0.94)
Triglycerides mmol/L, Mean (SD)	lbxtr, lbxtrsi	1.85 (2.17)	1.54 (0.82)
Creatinine mmol/L, Mean (SD)	NA	74.2 (26.85)	NA
Ferritin mmol/L, Mean (SD)	NA	109.48 (123.43)	NA

The combined patient cohort represents a group of 804 Type 2 diabetes mellitus patients. The EVAS data was originally published in [30]. The NHANES data is available in the official web portal of the National Health and Nutrition Examination Survey [31]

In the EVAS patient cohort, we examined data with regards to diagnosis of ophthalmic complications of diabetes at baseline, as well as their development over a period of up to 3 years after enrolment into the cohort study (Table 2). In both the EVAS and the NHANES cohorts male and female participants were represented in equal proportion (Table 1). In the EVAS cohort the mean age was 54, the median duration of type 2 diabetes, hyperlipidemia and hypertension was 10, 7 and 6.5 years, respectively. In the NHANES cohort the mean age of the participants was 60, and the data on the hyperlipidemia and hypertension was unavailable. In the EVAS cohort, at the baseline time point, cataract and retinopathy were diagnosed in 118 (41.0%) and 88 (30.4 %) patients, respectively. In the NHANES cohort, diabetic retinopathy was declared in 106 participants (20.5%), while the data on other ophthalmic complications was not collected. In this cohort, retinopathy declaration was done by answering “yes” to the question “Has a doctor ever told you that diabetes has affected your eyes or that you had retinopathy?” (dataset variable *diq080*).

**Table 2:** Summary statistics of ophthalmic complications in the EVAS observational cohort study

Variable	Summary
Cataracts, n (%)	118 (41.0)
Diabetic retinopathy, n (%)	88 (30.4)

The data was originally published in [30]

The summary biochemical characteristics of the patients are listed in Table 1. Similar to the earlier study [30], vascular functions of the EVAS patients were assessed. Table 3 provides the summary. The patients were characterized by measuring the concentrations of C-reactive protein (CRP), reactive oxygen molecules (ROM) and oxidized LDL (ox-LDL). Arterial stiffness was quantified by the pulse wave velocity (PWV), and the endothelial function was assessed with reactive hyperemia index (RHI).

**Table 3:** Vascular function measurements in the observational cohort study

Variable	Summary
CRP, mg/L Median (IQR)	1.45(0.7–3.8)
ROM, Median(IQR)	271(241–313)
BAP, $\mu$M Median(IQR)	2221(2061–2388)
Ox-LDL, IU/L Mean(Sd)	55.92(21.65)
CIMT, mm Mean(SD)	0.65(0.13)
LnRHI Mean(SD)	0.67(0.25)
Pulse Wave Velocity m/cm	8.35(1.74)

The data was originally published in [30]

### Computational modeling

#### Generalized metabolic flux analysis (GMFA)

Following the MFA theory, a metabolic network system described by a set of *M* metabolite species ($i = 1...M$) and *N* reactions/connections (enumerated $j = 1...N$) between the species, the stoichiometry matrix *S* (size $M \times N$) inherently characterizes the metabolic system and is assumed to be independent of time. A metabolic state is defined as the set of concentration $X_i$ of all *M* metabolites as well as of fluxes $v_j$ for all *N* reactions. The concentrations and fluxes changing in time (*t*) are related via the linear system of *N* flux balance equations:

1 $$\begin{aligned} \frac{d[X_i]}{dt} = \sum _j{S_{i,j}v_j(t)} \end{aligned}$$

Under a long-term smooth change along a trajectory between two metabolic states *A* and *B* is commonly quantified by the variable expressing the number of molecular transformations of one particular type that has to occur while the system transforms from state *A* to state *B*. By analogy with a single biochemical reaction progress, where this number is represented by a scalar variable $\xi$, termed the reaction extent, in a metabolic network it is represented by a reaction extent vector.

Similar to Eq. 1 describing changes in time *t*, fluxes and metabolite concentration changes can then be expressed with respect to changes expressed in the units of the extent $\xi$:

2 $$\frac{d[X_i]}{d\xi } = \sum _j{S_{i,j}v_j(\xi )}$$

A connection between the extent $\xi$, time *t* and metabolite concentrations $X^A$ and $X^B$ (at states *A* and *B*, respectively) can be described as follows:

3 $$X^B = X^A + \int _A^B \xi d\xi = X^A + \int _{t_A}^{t_B} \xi \frac{\partial \xi }{\partial t} dt = X^A + \int _A^B S v(\xi ) d\xi$$

Under these assumptions, changes of metabolite concentrations and metabolic fluxes are fully defined as functions of $\xi$. A single, continuous evolution path on the extent scale, includes all intermediate states of the system, including the starting state *A* and the final state *B*. If we observe an ensemble of distinct metabolic systems (e.g., organisms) observed at states *A* and *B*, their evolution on the extent scale $\xi$ would reflect their evolution in time. Thus the average metabolic state of the ensemble at a point on the scale $\xi$ would reflect the average time it took the systems of the ensemble to reach that state. Such process can be described as ergodic.

If in the system, whose metabolic state evolution is linear (as defined in Eq. 2) and there is a non-metabolic variable in that system, which is also linear in the selected extent coordinate $\xi$, the system of Eq. 2 can be extended with the given non-metabolic component without violations of the initial assumptions. Then, in such cases, the extent vector, the vector of metabolic fluxes and the stoichiometry matrix can be termed the generalized extent, the generalized fluxes and the generalized stoichiometry, respectively. The quantities of generalized fluxes obtained as the estimates to the observations of the individual’s measurements are termed here the GMFA digital twins.

For further details, please refer to Online Appendix A.

#### Digital twin construction

Digital twins were created using the GMFA methodology introduced in this study (see Online Appendix A for details). The construction and primary analysis of digital twins was implemented in Python (v.3.8) programming language.

**Fig. 1:**
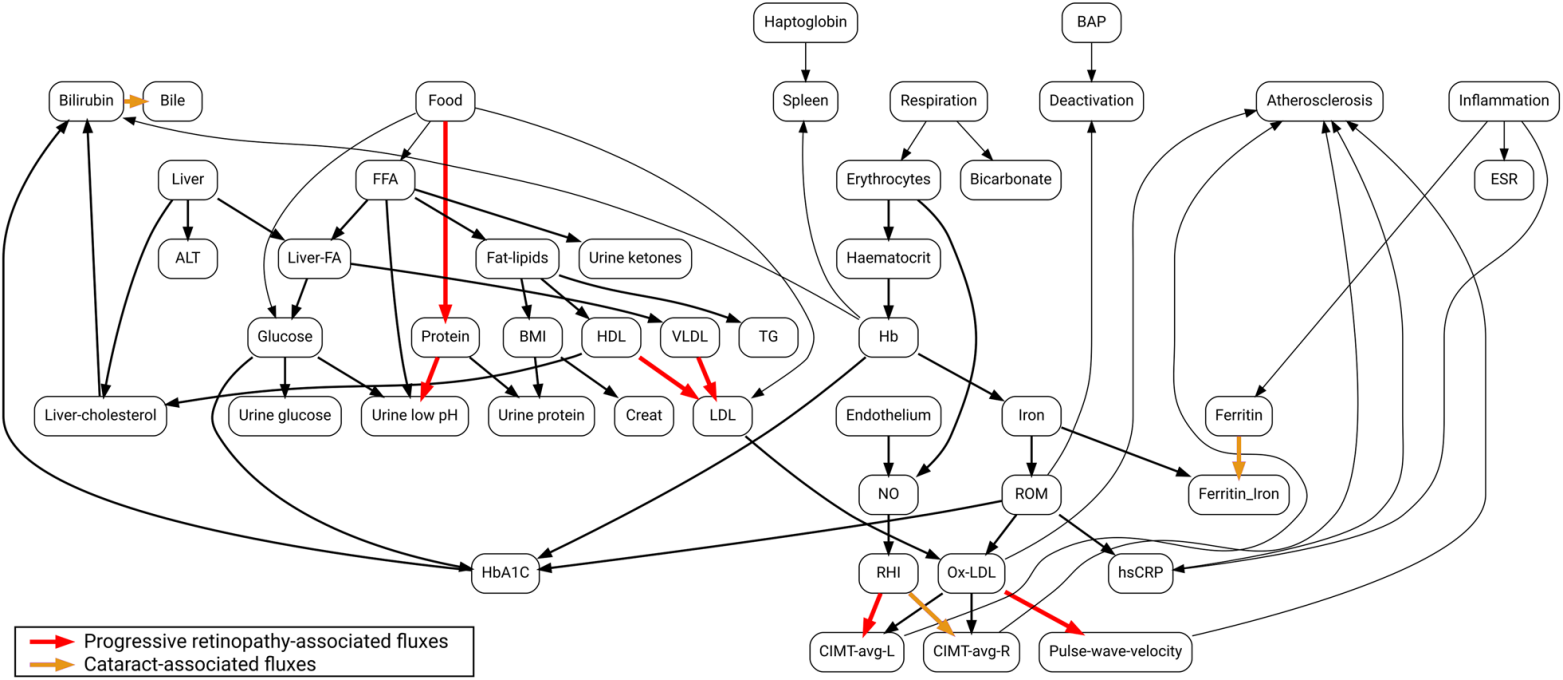
Metabolic flux map used in the simulation of diabetes health states. The map is a graphical representation of the extended stoichiometry matrix in Supplementary Table 1. Nodes represent metabolites and physiological parameters. Edges represent generalized fluxes. The fluxes statistically associated (P<0.01 by the Wilcoxon–Mann–Whitney test) with proliferative retinopathy and cataract are highlighted with red and brown, respectively

The metabolic map and the generalized stoichiometry matrix were designed to include the metabolites measured in the study, to quantify the fluxes through the major biochemical and physiological pathways implicated in diabetes (Fig. 1, Supplementary Table 1).

We extended the stoichiometry matrix by integrating in it the measured physiological parameters (see Appendix A and Supplementary Table 1). We illustrate this approach by finding a stoichiometric coefficient connecting the metabolic variable oxidized low-density lipoprotein (ox-LDL) with the physiological variable pulse wave velocity (Online Appendix B).

For each patient, a personalized digital model was constructed and initiated with all available metabolite and physiological readings measured from the patient. The missing data were imputed as population averages. Then, the best fit generalized flux vectors were obtained by the quadratic optimization procedure as the solution of the system of stoichiometric equations that minimizes the squared deviations between the vector of patient’s readings and the vector of the metabolite concentrations and physiological values in the model. Hereby, the patient’s initial data were used as soft constraints. Their respective values in the model were permitted to deviate within ± 20%. The constraints limiting the permitted flux values were introduced based on reference literature (see Online Appendix B for more details).

#### Distance metrics

The state of a given digital twin is defined by the vector of all generalized fluxes in the network at a given generalized extent. The proximity of two states can be assessed by applying a distance metric. We tested the Euclidean distance as well as the health state distance, a manifold-based metric (see Online Appendix A for the formulas).

#### The diabetes evolution trajectory

The initial point of diabetes type 2 progression (state *A*) was selected to represent a healthy individual with demographic characteristics similar to the studied population. The end-point state (state *B*) represented advanced type 2 diabetes, where common complications, such as hypertension and nephropathy are fully manifested. Each state was characterized by measured values of key metabolite concentrations and quantitative physiological parameters of vascular health (Tables 1, 3. ).

On the progress scale $\xi$ stretching between points *A* and *B*, we considered an individual patient’s health history as a smooth change of metabolite concentrations and physiological characteristics marked, in some cases, by developing diabetic complications.

### Data pre-processing

The EVAS dataset was collected as part of the earlier clinical study [30] as doctor’s notes in the electronic spreadsheets. The input variables, patient characteristics and the diagnoses, were compiled into a single comma-separated tabular file (CSV), using the R programming environment (R v.3.4.4). The variables were classified into numerical (measurements) and categorical (classification and diagnoses).

The NHANES dataset was obtained from the official website as a set of XPT (SAS export format) table files containing cross-sectional data arranged by year and by the variable group. The files were converted to CSV tables, using the R programming environment. The data were aggregated across all the years and the available variables and saved in a single CSV file. The variables were interpreted according to the official description of the tables as categorical or numerical. *NA* values were assigned to missing data.

### Statistical methods

All the statistical methods were implemented using the R programming environment (R v.3.4.4).

#### Wilcoxon–Mann–Whitney tests

Each component of the metabolite and flux vectors was then evaluated as an independent predictor of the patient classification by one of the following phenotypes representing diabetic complications detected by the sample collections time point: diabetic retinopathy or cataract. Statistical associations between each vector component and each phenotype were assessed using the Wilcoxon–Mann–Whitney non-parametric test with the null hypothesis of the vector components being equal between two groups patients corresponding to two phenotypic (disease) states. Multiple hypothesis testing bias was controlled via the false discovery rate assessment. The false discovery rate was calculated according to the Benjamini–Yekutieli procedure [32], and the P-values with FDR not exceeding 15% were reported.

#### Logistic regression models

To identify at the baseline and to predict the development of the ophthalmic complications at the follow-up time points, we employed the binomial logistic regression model, implemented in the R v.3.4.4 standard library as a Generalized Linear Model (the *glm.fit* function) [33].

The magnitudes and signs of individual’s generalized fluxes, obtained at the baseline time point were used as the inputs of the model. The diagnosis at the follow-up time was taken as the expected output to obtain the best logistic regression coefficients parametrizing the model.

The quality of the model was assessed using the Receiver Operating Characteristic (ROC) and the c-index (area under the curve, AUC), as implemented in the R package *pROC* [34, 35].

To further increase the specificity of the models, samples with the intermediate values of the calculated logistic function (the “twilight zone” exclusion) were removed from the analysis. We tested the results obtained from removing the samples from the following percentile ranges: 45 to 55, 40 to 60, 35 to 65, 33 to 67, and 25 to 75. At each range, we obtained ROC estimates as described above.

#### Resampling procedures

To balance the test design to ensure robustness, we sampled 50% of positive cases and an equal number of negative cases to train the logistic model We used the other 50% of the cases to test it. We repeated the sampling 50 times to obtain median estimates of the ROC curves.

#### Linear regression between the flux distance metric and the flux-based diagnosis estimate

To quantify the correlations between the health state distance metric and diagnosis of the patients, we applied a binomial logistic regression model in R v.3.4.4 [33]. We used the values of metabolic fluxes as the input variables of the model. The binary output variable was the diagnosis of the patient at the baseline time point. The parameters of the logistic function were fitted as the optimal weights of the extended metabolic fluxes minimizing deviation between the observed and expected outputs across the patient cohort. The diagnosis variable received the value of 1 if the patient was diagnosed with a particular syndrome (here, retinopathy and cataract) and 0 otherwise. Since the patient cohort contained unequal number of patients with and without diagnosis, we applied statistical resampling to obtain balanced design to train the logistic regression model. Mean values of the regression coefficients obtained after 10 resampling steps were used as a basis for subsequent evaluation of the patient’s diagnosis. Evaluating the parameterized logistic function on a particular patient resulted in the value between 0 and 1, estimating the expected likelihood that the patient’s diagnosis was positive. Across the patients, correlation between the value of the logistic function with the distance metric was evaluated with the Kendall’s $\tau$ coefficient and test statistic.

## Results

### Application of the GMFA framework to diabetes patients’ data reveals metabolic and physiological mechanisms associated with diabetic retinopathy and cataract progression

We considered ophthalmic complications of type 2 diabetes of our patient cohort [30] to explore metabolic and physiological pathways, using the proposed methodology. The results are presented in Tables 4 and 5 (see also Fig. 1).

**Table 4:** Statistically significant associations between individual metabolites and physiological variables and ophthalmic complications in type 2 diabetes patients

Metabolite	Phenotype	P	FDR
Pulse wave velocity	Proliferative retinopathy	1.3E−3	0.076
RHI		1.8E−3	0.099
CIMT-avg-R	Cataract	2.3E−5	2.0E−3
CIMT-avg-L		1.1E−4	8.7E−3
HbA1c		2.3E−3	0.11

The P-values were obtained by using the Wilcoxon–Mann–Whitney statistical test with null hypothesis that the median flux values are identical in two groups of patients: with the phenotype being present or absent. The FDR was calculated using the Benjamini–Yekutieli P-value adjustment method for multiple hypothesis testing [32]

**Table 5:** Statistically significant associations between the fluxes connecting metabolic and physiological variables and ophthalmic complications in type 2 diabetes patients

Flux	Phenotype	P	FDR
$Food \rightarrow Protein$	Proliferative retinopathy	2.4E−5	2.0E−3
$Protein \rightarrow Urine-pH$		2.4E−5	2.0E−3
$HDL + VLDL \rightarrow LDL$		9.7E−6	1.0E−3
$Inflammation \rightarrow Ferritin$		1.9E−4	0.01
$RHI \rightarrow CIMT-avg-L$		4.9E−4	0.027
$Ox-LDL \rightarrow Pulse-wave-velocity$		2.8E−3	0.096
$RHI \rightarrow CIMT-avg-R$	Cataract	1.1E−4	7.1E−3
$Bilirubin \rightarrow Bile$		2.3E−3	0.089
$Inflammation \rightarrow Ferritin$		3.5E−3	0.096

The P-values were obtained by using the Wilcoxon–Mann–Whitney statistical test with null hypothesis that the median flux values are identical in two groups of patients: with the phenotype being present or absent. The FDR was calculated using the Benjamini–Yekutieli P-value adjustment method for multiple hypothesis testing [32]

Analysis of metabolite concentrations and physiological measurements (the results are shown in Table 4) revealed that pulse wave velocity (PWV, P = 1.3e−3) and reactive hyperemia index (RHI; P = 1.8e−3) were significantly increased in diabetes patients with proliferative diabetic retinopathy. Similar observations were reported earlier [36–38]. Cataract presence was significantly associated with carotid intima-media thickness (CIMT; P = 2.3e−5 in the right carotid artery and P = 1.1e−3 in the left one) and glycated haemoglobin (HbA1c; P = 2.3e−3). Analysis of statistical associations with metabolic fluxes provided more information than metabolites and physiological parameters alone (results shown in Table 5).

In the case of diabetic retinopathy, we found that the rate of protein consumption and protein-dependent decrease of urine pH are the parameter significantly associated with the diagnosis (P = 2.4e−5). Studies report equivocal effects of protein consumption on diabetic retinopathy development [39]. At the same time, there is a consensus with respect to the role of high protein consumption in diabetic microvascular changes [40], which can also be observed in associations with diabetic nephropathy and has been reflected in clinical recommendations [41, 42]. Urine pH is considered an independent negative prognostic and progression indicator of type 2 diabetes [43]. At the same time, the ammonium ions concentration is considered a factor significantly affecting urine pH of diabetes patients [44]. Another flux of significance was conversion of high-density lipoprotein cholesterol (HDL) into low-density lipoproteins (LDL) localized in the liver (P = 9.7e−6). Unlike many other tissues producing cholesterol locally, for e.g., the retina, the cholesterol produced by the liver is transported via the bloodstream [45]. LDL and the HDL/LDL ratios are known as significant factors of diabetic retinopathy progression [45–47]. The flux quantifying the effect of oxidized LDL (ox-LDL) on PWV was also significant (P = 2.8e−3). LDL oxidation and lipid oxidation in general are important mediators implicated in retinopathy [46]. Iron and ferritin play an important role in oxidation reactions affecting diabetic retinopathy progression [48], in particular, by producing ox-LDL [49], and our results support that (P = 1.9e−4).

Development of cataract was significantly associated with the extended flux leading from the reactive hyperemia index (RHI) characterizing the vascular health state, to carotid intima-media thickness (P = 1.1e−4). Notably, we also found that the flux leading to induction of ferritin and the flux converting bilirubin to bile, were also associated with cataract development (P = 3.5e−3 and P = 2.3e−3, respectively). Both associations were not detected on the level of individual metabolites. Recently, evidence was found that blood bilirubin might be a compound protecting retina from degradation in diabetes patients [50–52].

Thus, we observed that with respect to specific clinical phenotypes, statistical results obtained with metabolic and physiological flux models are not contradicting the results obtained with metabolic and physiological variables alone. Moreover, flux models have the potential to provide more biomarkers characterizing the disease and to improve statistical power. The ability of flux models to describe additional details of long-term dynamics is complementary to the descriptive power of metabolites alone. These conclusions confirm the applicability of the computational framework provided by GMFA to address the practical needs of integrative biochemical, physiological and clinical data analysis for holistic assessment.

### The distance between metabolic health states quantifies the progression of vascular diabetic complications

We used the extent variable $\xi$ to measure the state evolution and disease progression. Since the states are defined in terms of generalized fluxes changing along the $\xi$ scale, the distance metric on that scale should be expressed as a function of generalized fluxes and needs to reflect both qualitative changes and quantitative differences observed upon transitions between any two states. Thus, irrespective of individual variations in time scales of disease progression, health state and the extent variable $\xi$ are expected to reflect disease-associated physiological changes observed in the generalized flux profiles across the patient cohort.

We analyzed the influence of choosing any of four definitions of the distance between health states:

- Diabetes duration
- HbA1c value evolution
- The Euclidean distance between the patient flux profiles
- The health state distance, the manifold-based metric

The results of this analysis are presented in Tables 6, 7 and 8.

**Table 6:** Correlation analysis of physiological parameters of diabetes complications with the health state progression extent metric and diabetes duration in diabetes patients

Parameter	Diabetes duration		Serum HbA1c		Euclidean distance		Health state distance	
	$\tau$	$P(\tau )$	$\tau$	$P(\tau )$	$\tau$	$P(\tau )$	$\tau$	$P(\tau )$
HbA1c	0.20	1.3E−06	1.00	3.3E−137	− 0.09	3.0E−02	0.14	6.5E−04
Reactive hyperemia index	0.00	9.6E−01	− 0.08	3.9E−02	0.09	2.2E−02	0.13	7.9E−04
Systolic blood pressure	0.12	2.0E−03	0.01	8.2E−01	0.08	3.9E−02	0.12	3.0E−03
Left-side CIMT	0.15	4.8E−04	0.05	1.9E−01	− 0.11	5.1E−03	0.07	7.0E−02
Average CIMT	0.14	7.1E−04	0.03	4.0E−01	−0.09	2.7E−02	−0.08	5.9E−02
Right CIMT	0.11	7.5E−03	− 0.02	6.5E−01	0.08	4.5E−02	− 0.11	9.3E−03
Patient’s age	0.26	1.2E−10	− 0.07	9.7E−02	− 0.10	1.7E−02	− 0.11	6.2E−03
Body mass index	0.01	8.3E−01	0.08	3.5E−02	0.09	2.9E−02	− 0.12	2.7E−03
Urine albumin/creatinine ratio	0.12	9.4E−03	0.25	1.9E−08	− 0.07	9.4E−02	− 0.12	7.5E−03
Pulse wave velocity	0.25	5.9E−10	0.10	9.6E−03	0.09	2.2E−02	− 0.13	1.3E−03

The analysis was done using the Kendall’s $\tau$ correlation

**Table 7:** Correlation analysis of physiological parameters of diabetes complications with the health state progression extent metric and diabetes duration in patients with diabetic retinopathy

Parameter	Diabetes duration		Serum HbA1c		Euclidean distance		Health state distance	
	$\tau$	$P(\tau )$	$\tau$	$P(\tau )$	$\tau$	$P(\tau )$	$\tau$	$P(\tau )$
Systolic blood pressure	0.07	3.1E−01	− 0.01	9.1E−01	− 0.14	5.1E−02	0.23	1.7E−03
Average CIMT	0.10	1.9E−01	0.15	4.7E−02	− 0.24	9.9E−04	0.22	2.7E−03
Left-side CIMT	0.08	3.1E−01	0.12	1.2E−01	− 0.15	4.4E−02	0.22	3.7E−03
HbA1c	0.15	4.6E−02	1.00	3.5E−42	− 0.18	1.5E−02	0.21	3.5E−03
Right CIMT	0.15	4.5E−02	0.08	3.0E−01	− 0.20	7.5E−03	− 0.17	2.6E−02
Patient’s age	0.23	2.5E−03	− 0.07	3.7E−01	− 0.17	2.0E−02	− 0.18	1.2E−02
Body mass index	− 0.08	3.0E−01	− 0.01	8.6E−01	− 0.18	1.1E−02	− 0.21	4.5E−03
Pulse wave velocity	0.10	1.6E−01	0.06	3.8E−01	0.16	3.2E−02	− 0.21	4.1E−03
Reactive hyperemia index	0.02	8.2E−01	− 0.05	4.8E−01	0.15	4.6E−02	− 0.23	1.7E−03
Urine albumin/creatinine ratio	− 0.03	7.2E−01	0.15	8.3E−02	− 0.21	1.3E−02	− 0.24	3.5E−03

The analysis was done using the Kendall’s $\tau$ correlation

When considering all diabetes patients (Table 6), we find that all four distance definitions deliver qualitatively similar results. Regardless along which descriptor the disease progression is measured, it strongly correlates with patient age, followed by PWV, HbA1c, CIMT values, and the urine albumin/creatinine ratio. Notably, the two flux-derived distance metrics showed significant, but consistently lower correlation coefficient values for these variables compared with diabetes duration and serum HbA1c. Yet more importantly, we observe additional significant correlations between the health distance metric and the disease progression hits; e.g., hits for RHI ($\tau = 0.13$, $P = 7.9e-4$) and BMI ($\tau = -0.12$, $P = 2.7e-3$) are not seen for the other metrics.

**Table 8:** Correlation analysis of physiological parameters of diabetes complications with the health state progression extent metric and diabetes duration in diabetes patients with cataract

Parameter	Diabetes duration		Serum HbA1c		Euclidean distance		Health state distance	
	$\tau$	$P(\tau )$	$\tau$	$P(\tau )$	$\tau$	$P(\tau )$	$\tau$	$P(\tau )$
Systolic blood pressure	0.18	3.9E−03	0.05	4.6E−01	− 0.12	4.6E−02	0.20	1.7E−03
HbA1c	0.19	3.1E−03	1.00	2.3E−56	0.13	4.2E−02	0.17	5.6E−03
Left-side CIMT	− 0.02	7.3E−01	0.03	6.1E−01	− 0.12	5.5E−02	0.14	3.2E−02
Average CIMT	0.00	1.0E+00	0.02	7.4E−01	− 0.10	1.3E−01	− 0.14	2.3E−02
Patient’s age	0.16	1.1E−02	− 0.12	5.5E−02	0.12	4.9E−02	− 0.15	1.5E−02
Urine albumin/creatinine ratio	0.14	5.0E−02	0.23	1.2E−03	− 0.14	5.1E−02	− 0.17	1.7E−02
Body mass index	0.07	2.9E−01	0.07	2.7E−01	− 0.16	9.1E−03	− 0.18	4.1E−03
Right CIMT	0.06	3.8E−01	− 0.05	4.8E−01	0.16	1.5E−02	− 0.18	5.5E−03
Pulse wave velocity	0.27	2.2E−05	0.12	6.5E−02	0.17	5.7E−03	− 0.19	2.1E−03
Reactive hyperemia index	− 0.02	8.0E−01	− 0.07	2.7E−01	0.17	5.7E−03	− 0.20	1.8E−03

The analysis was done using the Kendall’s $\tau$ correlation

However, qualitatively different performances of the four distance metrics are observed if we explore the development of diabetic complications. In Table 7 (correlation with diabetic retinopathy), as a trend, the correlation coefficients and their significance measured for the Euclidean distance and our flux distance metric are consistently better than those for diabetes duration and HbA1c value evolution. Strikingly, the flux distance metric outperforms the Euclidean distance in both absolute correlation and significance in all but three cases (average and right-side CIMT, patient age). The flux distance metric significantly correlates with all the parameters, while all other distance measures are not significantly associated with some of them. Diabetes duration correlated only with patient age ($\tau = 0.23$, $P = 2.5e-3$), CIMT ($\tau = 0.15$, $P = 4.5e-2$ for the right carotid artery) and HbA1c ($\tau = 0.15$, $P = 4.6e-2$), while the health distance metric correlated with all the parameters ($|\tau | \ge 0.17$, $P \le 2.6e$-2).

Results for the diabetic patients with cataracts (see Table 6) reveal the same pattern. We find that diabetes duration and HbA1c do not significantly correlate with several listed physiological parameters, whereas the two flux-defined distances do. Hereby, for patients with complications, the metric from Equation 6 performs better than the Euclidean distance in terms of absolute correlation and significance in all but one case (left-side CIMT).

**Fig. 2:**
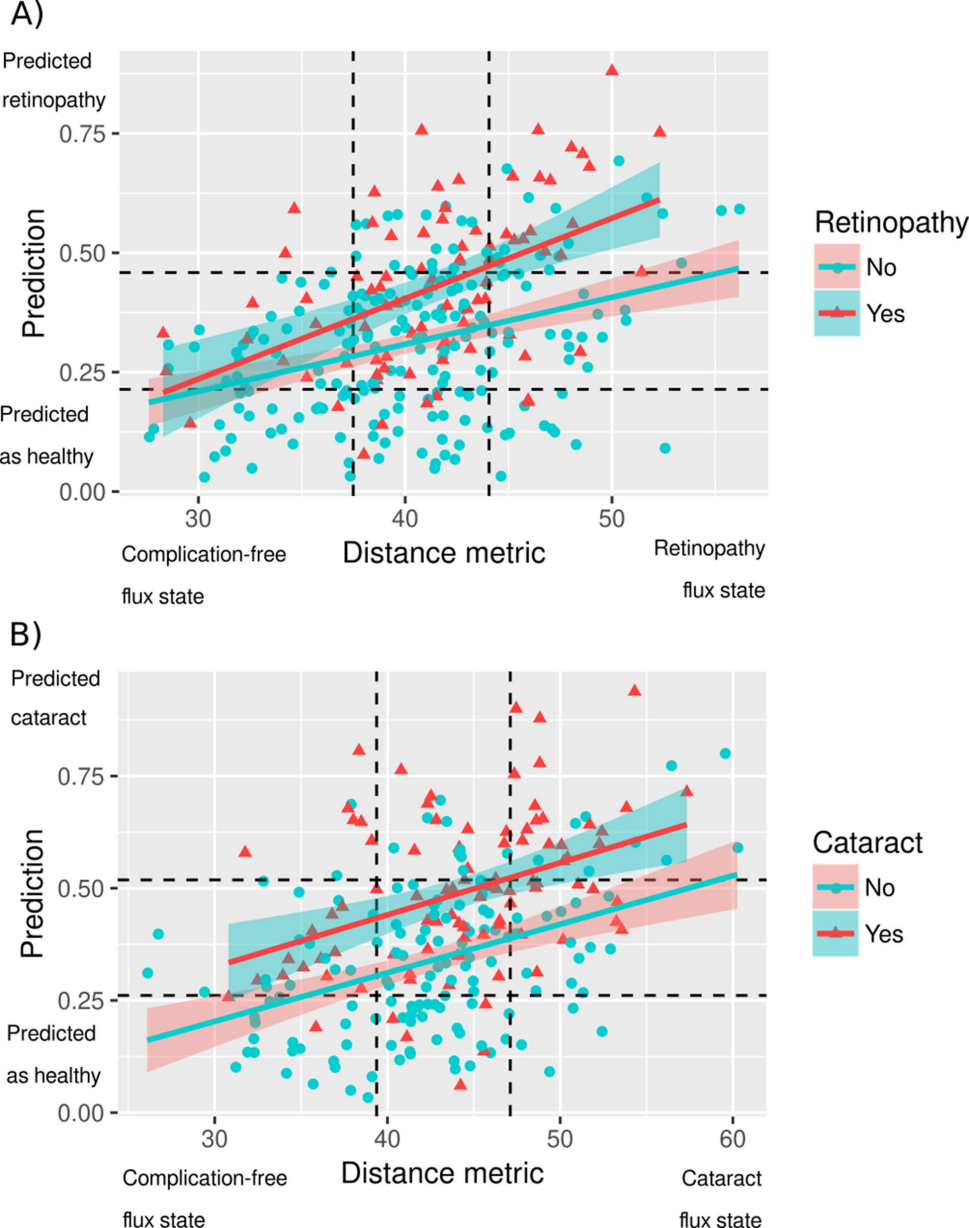
Correlation of the health state distance metric with diagnosis. For the diabetes patients diagnosed with retinopathy (**A**) and cataract (**B**) logistic regression model was parameterized to discriminate the present patient’s diagnosis, based on the computed values of the extended fluxes. This value is plotted along the vertical axis as the model-based diagnosis prediction. For each patient, we also calculated the distance metric of proximity of each individual patient to the health state without diabetic complications. The larger is the distance metric, the further is the estimated extent of patient’s progression towards a particular complication. The distance metric is plotted along the horizontal axis. We tested the hypothesis that distance of the patient’s health state progression and the predicted diagnosis, using the Kendall’s $\tau$ rank correlation coefficient. The results indicate significant statistical association between the two for both retinopathy ($\tau = 0.39$, $P = 8.0e-12$) and cataract ($\tau = 0.43$,$P = 6.6e-12$). The dashed lines shown the 25% and the 75% percentiles of the variables plotted along each of the axes. The patients with complications under the lower percentile boundaries in the lower axis could be interpreted as the ones whose generalized flux profiles are close to non-complicated diabetes. The patients without complications above the upper percentile boundaries in the lower axis could be interpreted as having generalized flux profiles are close to the profiles of patients with manifested respective diabetic complications

We tested whether the patients’ health state (defined by the generalized flux vector) can correlate with the trend towards an ophthalmic complication. As the output of the model on each patient, the value of the logistic function, ranging between 0 and 1, quantified the probability of the present patient’s diagnosis. This value is plotted against the flux distance metric in Fig. 2. We observe that the distance metric strongly correlates with the logistic function output for retinopathy ($\tau = 0.39$, $P = 8.0e-12$; see Fig. 2A) and cataract ($\tau = 0.43$, $P = 6.6e-12$; see Fig. 2B). Across all patient groups, with and without diabetic complications, the positive correlations indicate the trend of increasingly poor identification of the diagnosis for the patients, whose flux profiles are further away from the complication-free status.

Using the distance metric of the flux profiles and the generalized flux-driven syndrome identification, patients with high and low risk of complications can be classified into the respective high- and low-risk groups. Here, we assigned the patients to the high-risk group by their proximity to the diagnosed syndrome state if they fall into the upper quartile simultaneously by (i) their logistic function value and (ii) the distance (see the distance metric above) between their current health states (flux profiles) and the complication-free state. This group corresponds to the top-right quadrant in Fig. 2A and B. The patients of the low-risk group were defined by the values of their logistic function and the distance metric corresponding to the lower quartile (the bottom-left) quadrant in Fig. 2A, B), corresponding to the health states with the highest rates of diabetes complications. Confirming the results of the correlation analysis, the low-risk and the high-risk patients demonstrated a strong statistical association with their actual diagnoses of retinopathy ($P = 5.1e-5$) and cataract ($P = 1.3e-5$), as shown in Table 9.

**Table 9:** Association of the patient risk group with diagnosed diabetic retinopathy and cataract

Syndrome	Risk group	Patients without diagnosis	Patients with diagnosis
Retinopathy	Low risk	27	2
	High risk	17	20
Fisher’s exact test $P = 5.1e-5$			
Cataract	Low risk	22	2
	High risk	9	19
Fisher’s exact test $P = 1.3e-5$			

Patients were considered low risk when their flux profiles and their flux-driven syndrome prediction are within the lower quartiles (the bottom left quadrant shown in Fig. 2). The patients, whose flux profiles and their flux-driven syndrome prediction are within the higher quartiles (the top right quadrant shown in Fig. 2), were considered high risk. Both diagnoses demonstrate a highly significant co-incidence with the risk groups ($P \le 5.1e-5$, Fisher’s exact test)

**Fig. 3:**
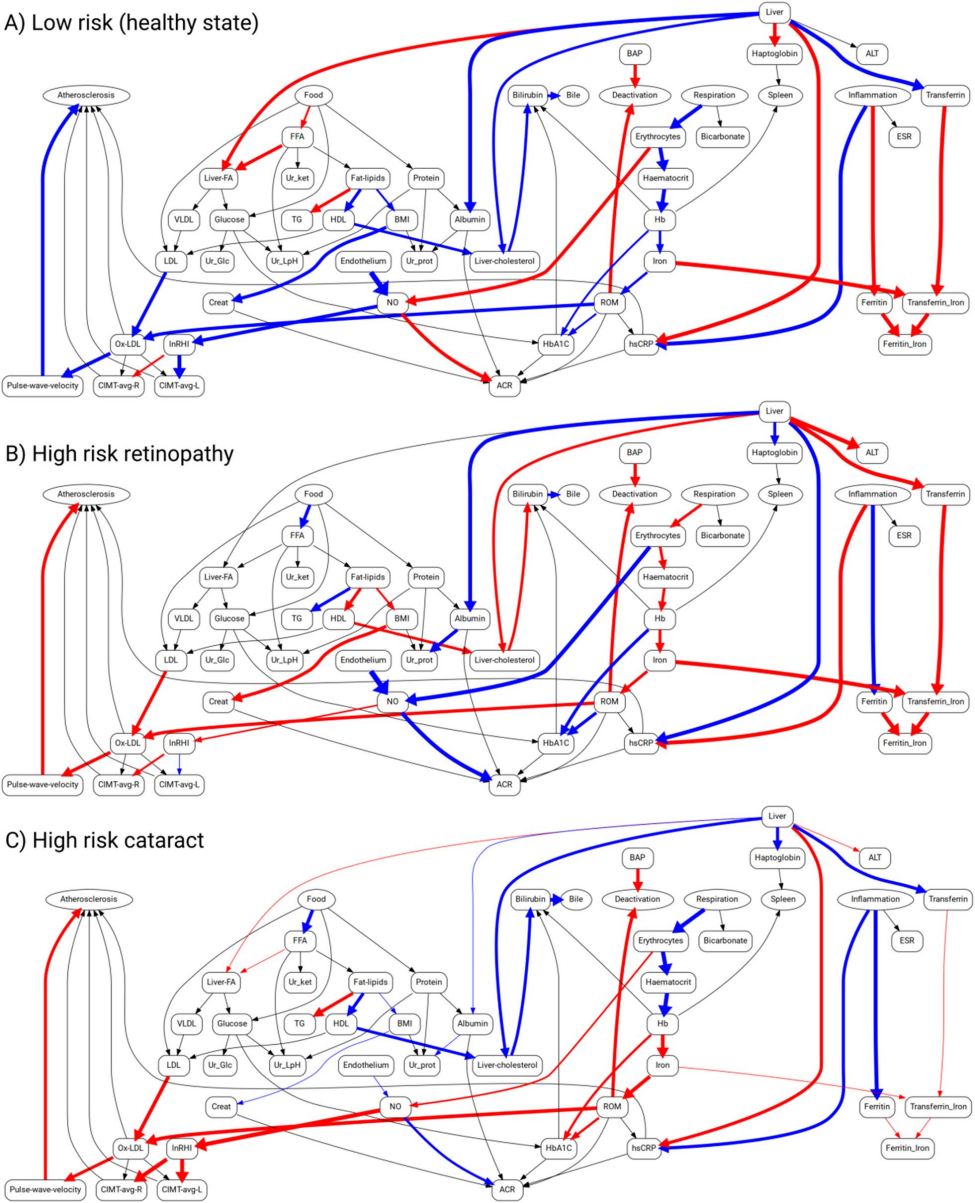
Graphical representation of the flux states of the diabetes patients with low risk of ocular complications (**A**), high-risk retinopathy (**B**) and high-risk cataract (**C**). The flux states were obtained by calculating the median flux magnitudes within the risk groups. The magnitudes are displayed normalized relative to the median across the entire studied population of diabetes patients. The blue and red edge colors in the graph represent the median flux values lower or higher than the population median, respectively. Edge thickness represents the relative magnitude of difference between the absolute values of the group median and the population median value of a particular flux. The groups are defined according to Table 9 and Fig. 2 as follows: **A** low-risk group for retinopathy and cataract (the lower quartile in Fig. 2A and 2B); **B** high risk retinopathy group (the upper quartile in Fig. 2A); **C** high risk cataract group (the upper quartile in Fig. 2B)

To characterize the high- and low-risk groups, we calculated their median flux profiles and displayed them as graphs with edge weights (representing the fluxes magnitudes) proportional to the normalized deviation from their median values in the entire patient cohort. These values are displayed in a graphical form in Fig. 3. We observe that the flux rates immediately upstream and downstream ox-LDL are commonly decreased in the low-risk group, relative to both high-risk groups. High-risk retinopathy patients could be differentiated from the high-risk cataract patients by increased fluxes upstream creatinine, increased liver cholesterol metabolism, and increased haemoglobin-related fluxes. High-risk cataract patients are specifically characterized with increases in haemoglobin glycation fluxes leading from ROM to HbA1c and with hs-CRP induction.

Thus, we can conclude that the distance between a patient’s flux profile and a typical flux profile of a complication-free diabetes patient is indicative of the degree to which a particular diabetic complication is (or potentially will be) manifested in a given patient.

Together these results show that, for a given diabetic complication, it is possible to find such a disease progression metric that would better correlate with the evolution of key progression characteristics than direct clinical parameters, such as diabetes duration or HbA1c values. Further analysis of the flux profiles may uncover the mechanisms underlying syndrome development.

### Digital twins can indicate the presence of ophthalmic complications of diabetes at the baseline and predict their development 3 years in the future

**Fig. 4:**
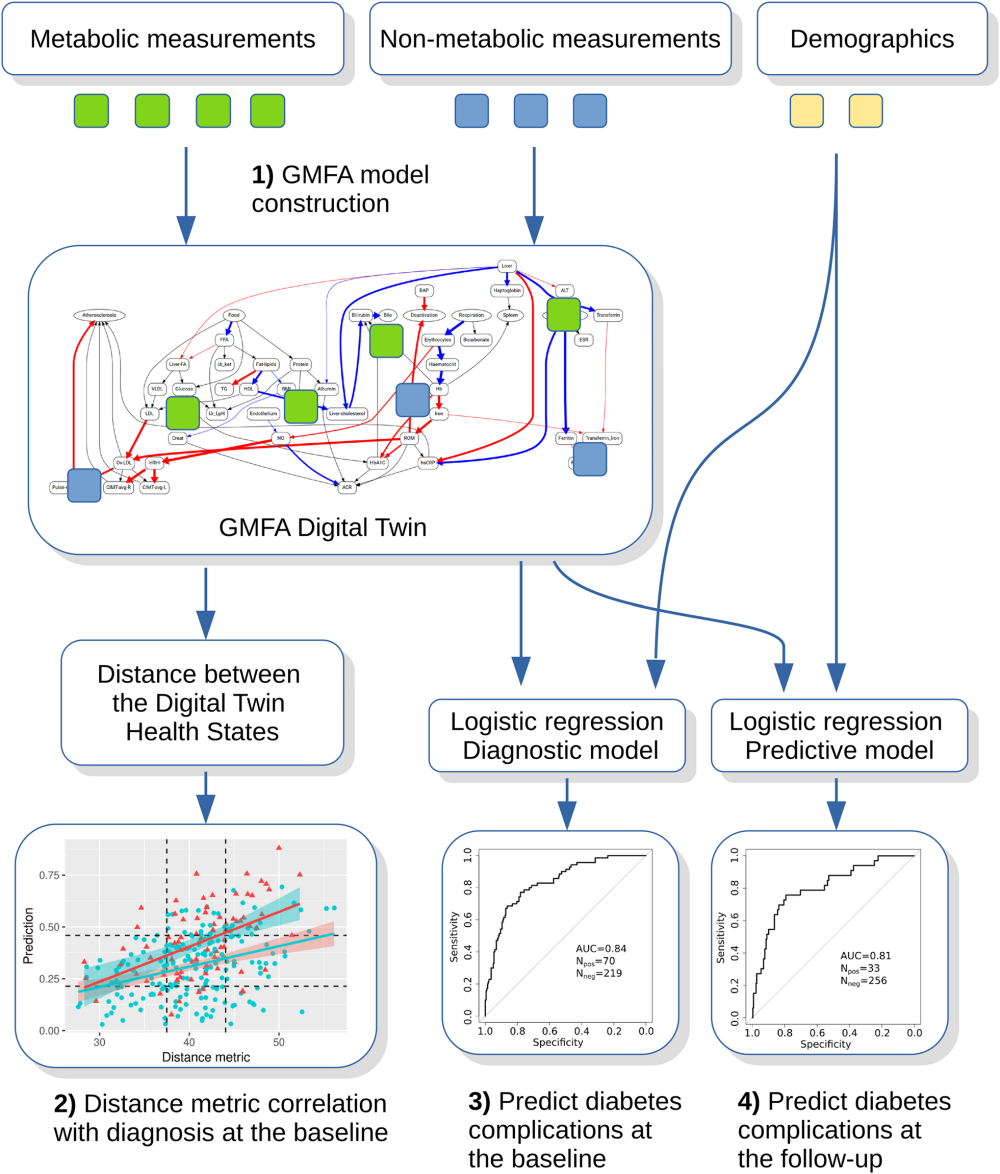
GMFA Digital Twins construction and evaluation. Metabolic and non-metabolic physiological variables were obtained from T2DM patients. The GMFA methodology was applied to construct digital twins representing individual patients’ health states at the baseline time point (1). The distance between the health state of the patient to the advanced disease state correlates with the risk of developing T2DM complications (2). By combining the GMFA digital twin profiles with the demographic data (age, diabetes duration) we constructed logistic regression models, which can identify patients with T2DM complications (3) and the patients who will develop them in the future (4)

To explore the potential of using the GMFA-based digital twins as diagnostic and predictive tools, we used them as inputs of logistic regression models for detection existing and predicting future occurrence of ophthalmic complications of type 2 diabetes in the EVAS dataset (see Table 2 for summary statistics). The analysis schema is shown in Fig. 4.

**Fig. 5:**
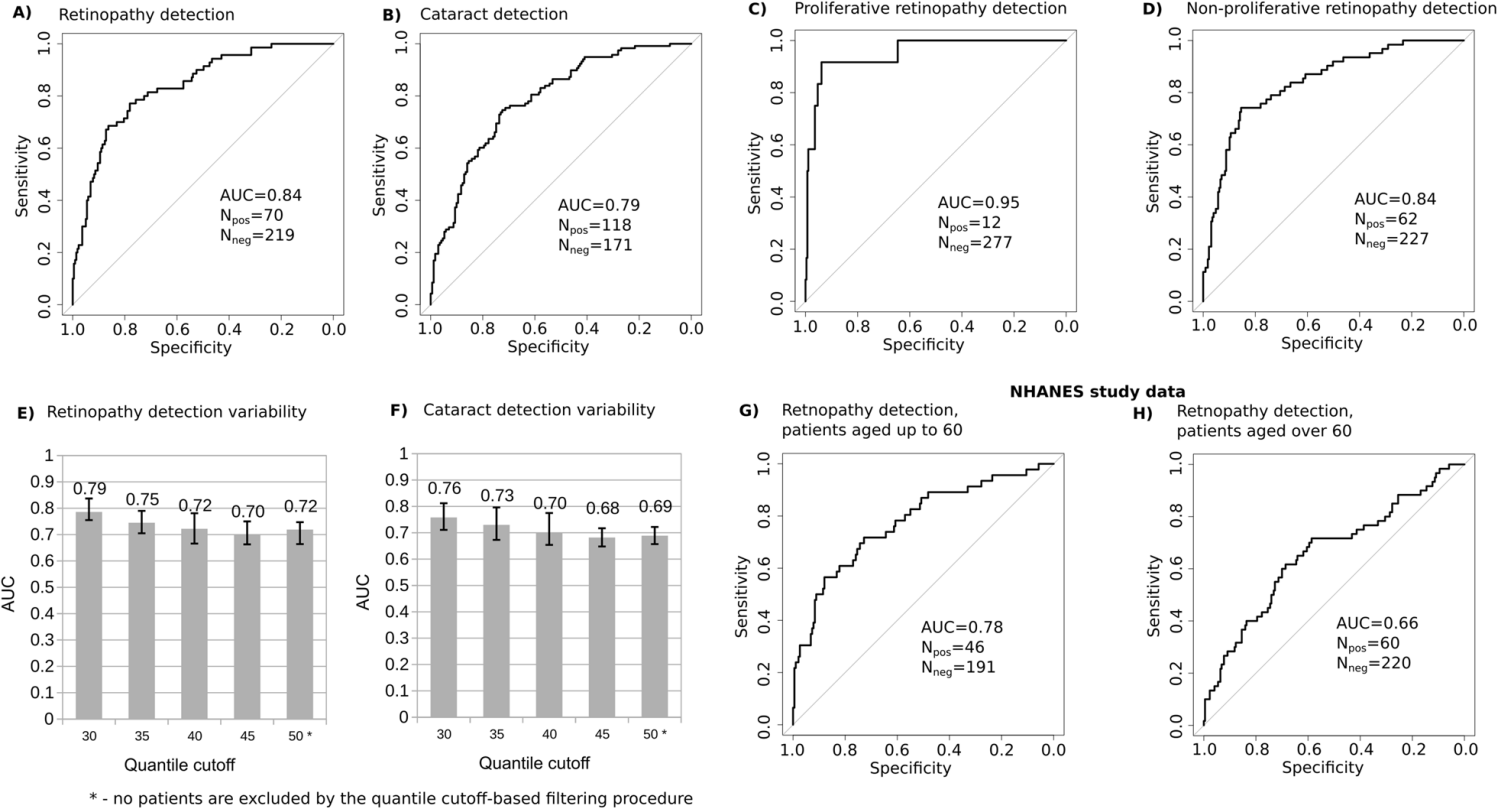
Performance of GMFA-based logistic regression models in identifying T2DM patients with present ophthalmic complications in the EVAS patient cohort and their validation in the NHANES cohort. Logistic regression models were built using the generalized fluxes (GMFA), patient’s age and diabetes duration as the input variables. The cross-sectional data from the EVAS patient cohort was used to evaluate the performance of the algorithm in detection of ophthalmic complications based on biochemical and physiological inputs. The regression models output the probability of the patient having diabetic retinopathy (**A**) or cataract (**B**). The retinopathy cases were further sub-classified into proliferative (**C**) and non-proliferative (**D**) subtypes. The models predicted diagnoses made at the baseline time point. The performance of the models is assessed with the area under the ROC curve (AUC). Variation in the AUC values for retinopathy (**E**) and cataract (**F**) was assessed via resampled training and testing datasets (50% positive rate in each). Median AUC values and the IQR-based confidence intervals are reported for 50 resampling iterations per each AUC estimate. The tolerance to poorly discriminated cases was tested by filtering the patients of the intermediate phenotypes by the health state distance and risk prediction logistic function (see Fig. 2 and the Results section for details). Percentile-based exclusion of patients with the intermediate phenotypes resulted in a slightly improved performance of the predictive models: from AUC 0.72 (retinopathy) and AUC 0.69 (cataract) with no filtering (Quantile 50* cutoff) to AUC 0.79 (retinopathy) and AUC 0.76 (cataract) when retaining 60% of patients (Quantile 30 cutoff) and filtering out the remaining 40% of patients with intermediate phenotypes. Retinopathy detection performance was validated by applying the GMFA models to the cross-section data from the NHANES patient cohort (**G**, **H**). For the subgroup of the NHANES patients with diabetes history duration within the range similar (AUC 0.78) to that of the EVAS cohort (**G**), the AUC value was similar to that of the EVAS cohort (**A**). For the NHANES patients with diabetes history spanning longer than 25 years, the AUC dropped to 0.66 (**H**)

The logistic regression models could identify in the population patient having retinopathy (AUC 0.84, SN = 80%, SP = 71%) and cataract (AUC 0.79, SN = 80%, SP = 62%). The results are reported in Fig. 5 (A and B, respectively). Sub-classifying all the retinopathy cases into proliferative and non-proliferative subtypes resulted in an increased performance for each of subtype: AUC 0.95 (SN = 92%, SP = 94%) for proliferative retinopathy and AUC 0.84 (SN = 80%, SP = 70%) for non-proliferative (Fig. 5C, D). To ensure the robustness of our results, we carried out the analysis by: (1) statistical resampling (50 iterations), (2) balancing the training and the testing set design to provide equal number of positive and negative cases. The results are shown in Fig. 5E and F.

Our analysis of relationship between the patient’s health state distance and the ability of the logistic model to identify the patient’s risk group (Fig. 2) suggests that there are patients with intermediate metabolic phenotype, whose classification into the risk groups is difficult. We took into account the potential impact of this intermediate sub-population on the performance metrics. We varied the fraction of patients with intermediate phenotypes in the training and the testing datasets by iteratively selecting only the patients whose distance metric and logistic function value (see Fig. 2) were either higher or lower than a given quantile value. This cutoff quantile value was iterated in the range from the 30th/70th percentile (40% of the patients with intermediate phenotypes excluded) to the 50th (no patients were excluded). At each iteration we quantified the observed AUC values. The results shown in Figs. 5E and 5F demonstrate that the reported median AUC values across all the tested scenarios are within the confidence range ($\pm 1 IQR$).

**Fig. 6:**
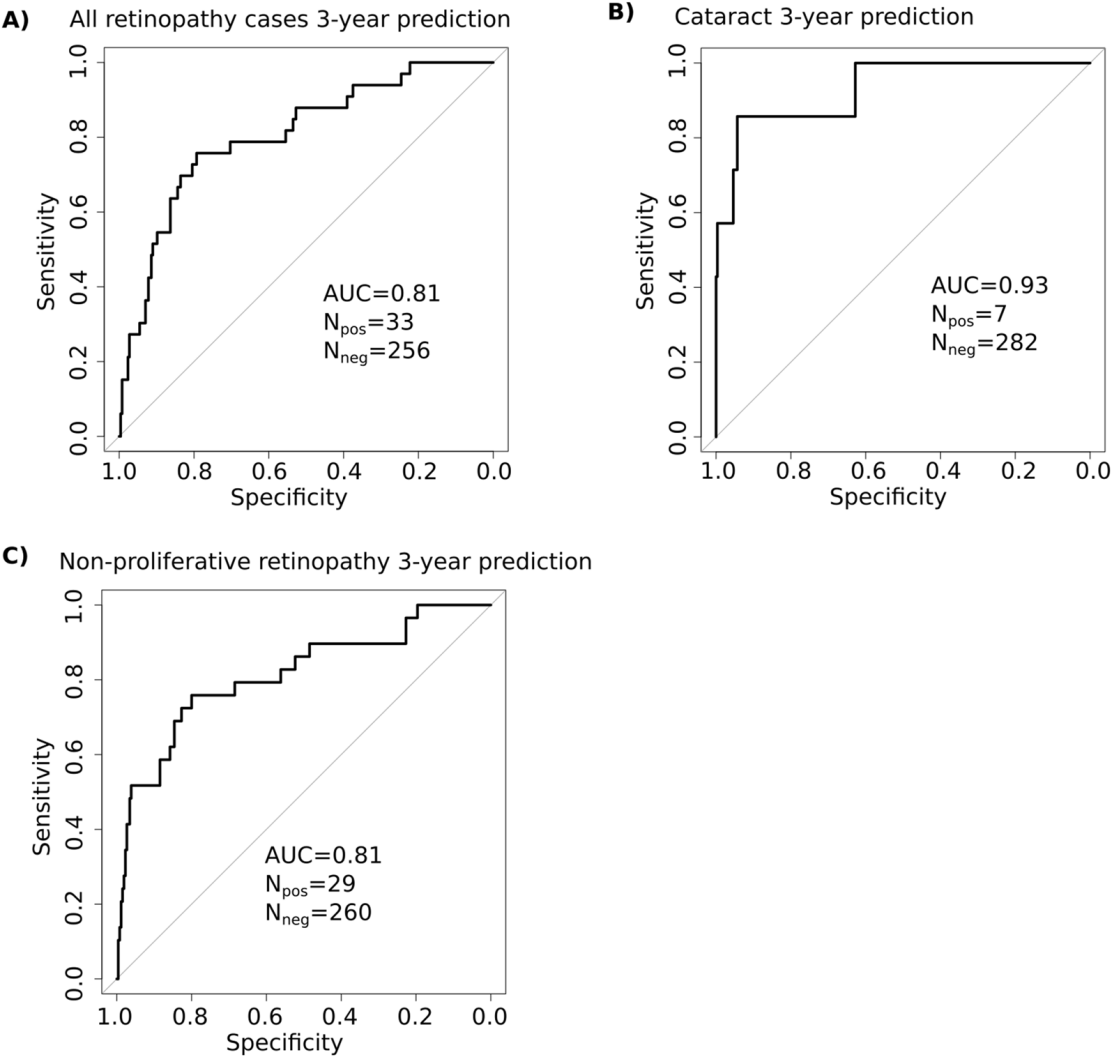
Performance of GMFA-based logistic regression models in predicting development of ophthalmic complications in T2DM patients within 3 years. Logistic regression models were built using the generalized fluxes (GMFA), patient’s age and diabetes duration as the input variables, at the baseline. The regression models output the probability of the patient to develop any subtype of retinopathy (**A**), cataract (**B**), or a non-proliferative retinopathy. The retinopathy cases were further sub-classified into proliferative (not shown) and non-proliferative (**C**) subtypes within 3 years after the baseline time point

To validate our findings in an independent patient cohort, we analyzed the National Health and Nutrition Examination Survey (NHANES) dataset, which included patient survey and measurements data collected across the United States [31]. From the NHANES data study, we selected a total of 517 subjects diagnosed with T2DM diabetes and characterized with the set of parameters matching those of the Singapore EVAS dataset (Appendix C, Supplementary File). Summary statistics indicated that the two populations were comparable with respect to most of the parameters (Table 1). The differences were observed in ethnicity, age distribution and incomplete information on the hypertension and hyperlipidemia in the NHANES cohort. Moreover, the rate of retinopathy in the NHANES cohort was twice lower than the EVAS cohort (20.5% vs 41%, respectively). When we reproduced identification of patients diabetic retinopathy in the NHANES dataset, using the logistic regression model similar to that of the EVAS dataset, we observed a markedly lower performance in NHANES (AUC 0.67). To test if the drop in performance was due to a lower homogeneity of the NHANES population, compared to EVAS, we separated the NHANES patients into two subgroups: one tightly matching EVAS patients by age (237 patients aged up to 60 y.o) the remaining subgroup containing more dissimilar patients (280 patients aged above 60). The logistic regression model in the first group demonstrated the performance close to that in the EVAS cohort (AUC 0.78, Fig. 5G). At the same time, the performance in the second group remained relatively low (AUC 0.66, Fig. 5H). These results indicated that, despite the disproportion in the retinopathy cases across the multi-ethnic populations, a good reproducibility of our methods can be achieved when key patients characteristics, such as age, are matched across the cohorts.

Having demonstrated the evidence of GMFA-based digital twin models indicating the presence of T2DM ophthalmic complications, we tested if the models can be used to predict development of these complications in the future, within 3 years from the baseline time point. The results are presented in Fig. 6.

We found that the GMFA models can predict all retinopathy (AUC 0.81, SN = 78%, SP = 70%, Fig. 6A) and cataract (AUC 0.93, SN = 87%, SP = 95%, Fig. 6B) cases. For non-proliferative retinopathy alone, we observed a slightly better performance (AUC 0.81, SN = 79%, SP = 70%, Fig. 6C). For proliferative retinopathy the analysis could not be performed due to the insufficient number of patients (4 patients) in this group.

## Discussion

Over the past 30 years, numerous systems biology based tools have been developed in the academia and been used in the pharmaceutical and biotechnology industry, for example, for optimization of fermentation processes. Metabolic flux analysis (MFA) is a flexible method of systems biology that have been tested in applications that span from bacterial models to higher eukaryotes [19–23]. However, these methods, which were based on unicellular biological models, were not easily scalable to tissues and organs. In particular, modeling human physiology in a clinical context proved to be extra challenging.

Precision medicine and digital health initiatives are driving the adoption of advanced computational tools for holistic analysis and interpretation of individual patients’ physiological and health states. At the same time, clinical science is trending towards a focus on an integrative picture of personalized health. Today, a holistic assessment of a person’s health state implies integration of detailed profiles of multiple physiologically inter-connected subsystems: metabolism, cellular signalling, immune responses, nervous system, body structure and microbiome. At present, there is no methodological framework to unify quantitative modelling of all these components.

In the present study, we describe the Generalized Metabolic Flux Analysis (GMFA). The combined impact of several technical innovations and novel concepts of GMFA enables the computational simulation of complex, clinically relevant networks. The critical points are:

- By pooling metabolites and fluxes along the biological processes and mechanisms, we both keep the biological logic of the systems and, at the same time, greatly simplify the network, making it much more coarse-grained.
- We further enhance the information content of the model network by mapping non-metabolic clinical modalities and measured clinical laboratory parameters onto the network.
- We analyze the system’s trajectories along the disease extent progression coordinate, rather than the time scale. In this way, the progression of various patients along the path from health to the manifested disease state becomes comparable.
- We calculate the system fluxes by quadratic optimization using the clinical readings as soft constraints. The optimality of the system’s profile in the space of generalized fluxes is formulated as the best fit ensuring the minimal squared difference between the observed measured variables and their values predicted from the given flux solution under constraints.

By applying GMFA to accessible clinical data we create descriptive and predictive personalized mathematical models of an individual patient’s metabolic state. A digital twin can be defined as a mechanistic numerical model of a particular patient calibrated to the individual’s phenotypic and clinical data at a particular time point. Thus, GMFA is used as a novel approach for creating digital twins based on evaluating observed metabolic and physiological data.

Within the GMFA framework, we analyzed two cohorts of diabetes type 2 patients, EVAS [30] and NHANES [31].

We built a coarse-grained metabolic map (Fig. 1) that includes non-metabolic edges related to PWV, CIMT, RHI and other clinical characteristics. We quantified the generalized metabolic fluxes in the system individually for each patient. Our correlation analysis demonstrates that application of GMFA reveals critical mechanisms associated with diabetic retinopathy and cataract progression (Tables 4, 5; Fig. 1). For example, we found that, in the course of retinopathy, chronic changes in the PWV are mediated via LDL oxidation stimulated by ferritin or that fluxes involving RHI, CIMT, ferritin and bilirubin are associated with cataract development. Further we found that distances between the metabolic health states of patients quantify the progression of diabetic vascular complications (Tables 6, 8; Fig. 2) and that the digital twins can be used for the prediction of their outcomes (Fig. 5).

The observed associations correlate with recent clinical and experimental studies reporting similar conclusions [45, 46, 48, 49]. In addition, our analysis supported earlier studies on the mechanisms relating nitric oxide production, reactive hyperemia index and atherosclerosis [53–56].

Thus, the GMFA method provides mechanistic insights into disease progression along a path in the health states space and allows us to delineate subgroups of patients that can be predicted to develop diabetic eye complications 2. This allowed us to build predictive model that can infer the present phenotypic state of the patient (the diagnosis made by the ophthalmologist) from the information on the patient’s metabolic dynamics provided by GMFA (Fig. 5). We also demonstrated that our models can predict development of ophthalmic complications in T2DM patients within 3 years from the baseline (Fig. 6). We processed the NHANES patient data with the same computational model developed for the EVAS data analysis without any further adaptation and we obtained very comparable results despite the great differences between the two cohorts (geographic location, ethnicity, age, etc.).

Our GMFA based method for creating digital twins as representations of health states that vary on the scale of the progress extent, has the advantage of relating the information obtained in a cross-sectional study of the population with evolution of health state in time. In the future, this approach may be further developed to evaluate different health state transitions with respect to reversibility. This would bring a novel perspective on options for chronic disease management.

In the last decade, there has been an upsurge in the use of data-driven machine learning models for the prediction of clinical outcomes. Machine learning provides prediction on outcomes of complex biological processes by ploughing through databases of inputs (exposures) and outputs (outcomes) for a given problem. These models bypass the need to understand complex mechanisms. In contrast, mechanistic modeling involves the generation of novel hypotheses for causal mechanisms that are generated through clinical observations in the datasets. A mechanistic model obtained by fitting its parameters to the available observations, would complement data-driven analyses by reducing the requirements for the data set volumes and compensating for occasional incompleteness of observations. The GMFA methodology described here, can be a candidate for this role. The advantages of similar systems biology methods can be found in integrating multi-level biological systems information, from genomics to proteomics [21, 29]. This provides future opportunities to use the GMFA as a framework for clinical systems medicine.

Our present study revealed several limitations. Despite the GMFA methodology is able to model medium- and large-scale metabolic networks, in the clinical setting only small-scale models can be practically applied, since a typical biochemical analysis includes only a small number of common biochemical tests. The available data sets included only up to two longitudinal data points per patient, often collected at an interval of a few years. Having a larger number of longitudinal data points would allow us test the linearity of the disease progression extent, one of the key assumptions of the analysis. Moreover, such design would allow us to test the ergodicity of the generalized fluxes. If the generalized fluxes are indeed ergodic, as predicted from the GMFA equations, this methodology could in the future be used to utilize large volumes of cross-sectional data as sources of information on longitudinal changes in the metabolism of patients in homogeneous populations. Despite many of diabetes patients receive medications, we were unable to effectively use this information, since the variation in the treatment regimes was great, while our patient cohorts were relatively small. This did not allow us to stratify our patients by treatment type into smaller subgroups, while having enough patients in each subgroup to achieve statistically significant conclusions.

Overall, our work demonstrates an example of using metabolic and physiological data to construct predictive digital twin models of patients from routinely accessible clinical data. We provide a novel analytical framework, which opens up possibilities for the elucidation of disease mechanisms in personalized health assessments. The GMFA approach was applied to modeling health states in diabetes patients and showed potential to predict the development of ophthalmic complications in patients with diabetes.

With further development and validation, the GMFA approach could be applied in the clinical setting for patient risk assessment. In the future, we plan to use our methods across a broad range of patient cohorts, phenotypes and diseases. A potential area of application of this methodology is in the stratification of patients in the process of population screening.

## Conclusion

The Generalized Metabolic Flux Analysis method described here aims to apply systems biology analysis principles to small- and medium-scale metabolic profiles, such as those obtained in clinical settings. The key novelty making the approach suitable for future clinical practice is building best-fit personalized constraint-based computational models (digital twins), which quantify the expected rates of inter-connected metabolic processes, based on a single time point data input. We validated this approach by generating the GMF digital twins from the biochemical and physiological data of type 2 diabetes patients. This allowed us to characterize the present metabolic states of individuals and to predict the health state progression into development of ophthalmic complications. The predictive performance in multiple patient cohorts, measured as ROC-AUC was in the range 0.79–0.95.

### Supplementary Information

Below is the link to the electronic supplementary material.

Supplementary file1 (PDF 192 kb)

Supplementary file2 (PDF 94 kb)

## Data Availability

Additional materials related with the study are available upon request to the authors.

## Abbreviations

MFA: Metabolic flux analysis
GMFA: Generalized metabolic flux analysis
M–M: Michaelis–Menten
PWV: Pulse wave velocity
LDL: Low-density lipoprotein cholesterol
ox-LDL: Oxidized low-density lipoprotein cholesterol
HDL: High-density lipoprotein cholesterol
CRP: C-reactive protein
ROM: Reactive oxygen molecules
RHI: Reactive hyperemia index
DHAP: Dihydroxyacetone phosphate
FDP: Fructose 1,6-bisphosphate
GAP: Glyceraldehyde-3-phosphate
PYR: Pyruvate
CIMT: Carotid intima-media thickness
HbA1c: Glycated haemoglobin
BMI: Body mass index
SBML: Systems biology markup language
SBGN: Systems biology graphical notation
