# Supplementary material for: Generalized metabolic flux analysis framework provides mechanism-based predictions of ophthalmic complications in type 2 diabetes patients
Source: Health Inf Sci Syst. 2023 Mar 29;11(1):18. doi: 10.1007/s13755-023-00218-x (PMC10060506; doi:10.1007/s13755-023-00218-x)
Supplement: Supplementary file 1 — Supplementary file1 (PDF 192 kb) [file 13755_2023_218_MOESM1_ESM.pdf]

Supplementary materials

August 30, 2022

Appendix A. Extension of the MFA framework

The extent variable quantifies metabolic system's state progression

Within the MFA framework, a **metabolic system** is described by a set of M metabolites (enumerated $i = 1...M$) and N reactions/connections (enumerated $j = 1...N$) between nodes in the metabolic network. A **metabolic state** is defined as the set of concentrations X_i of all M metabolites as well as of fluxes v_j for all N reactions. Concentrations and fluxes are related via the flux balance equation:

$$\frac{d[X_i]}{dt} = \sum_j S_{i,j} v_j(t) \tag{1}$$

Here, S is the stoichiometry matrix with the size $M \times N$. S inherently characterizes the metabolic system and is typically assumed to be independent of time and metabolic state. In such a condition, equation system 1 is linear and constrained linear optimization techniques can be used to find the best fit values of metabolic fluxes.

When we consider a metabolic system undergoing a long-term smooth change along a trajectory between states A and B, this gradual change of the system's state is commonly quantified by the variable expressing the number of molecular transformations of one particular type that has to occur while the system transforms from state A to state B. This variable is termed the **reaction extent** ξ. Real-life chemical systems consist of a large, but finite number of discrete molecules, and their minimal unit of the extent ξ is a single molecular transformation. However, in most real-world chemical and biochemical applications, the number of molecules is so large that it is impractical to trace reaction extent by individual molecules. Therefore, thermodynamic laws apply to this system, and the reaction extent ξ reads as a concentration change in a scalar or vector form. Its evolution can be analyzed as a smooth function of ξ (instead of as a function of time) along with thermodynamic variables.

Similar to Equation 1, fluxes and metabolite concentration changes can then be expressed in the differential form:

$$\frac{d[X_i]}{d\xi} = \sum_j S_{i,j} v_j(\xi) \tag{2}$$

Thus, a connection between the extent ξ, time t and metabolite concentrations X^A and X^B (at states A and B, respectively) can be calculated as follows:

$$X^B = X^A + \int_A^B \xi d\xi = X^A + \int_{t_A}^{t_B} \xi \frac{\partial \xi}{\partial t} dt = X^A + \int_A^B Sv(\xi) d\xi \tag{3}$$

We assume that changes of metabolite concentrations and metabolic fluxes are fully defined as functions of ξ. A single, continuous evolution path includes all intermediate states of the system, including the starting state A and the final state B. If all fluxes are considered reversible, any two states can be transformed into each other in both directions along the evolutionary path. Such systems are termed ergodic, i.e., transformations can be analyzed along a trajectory in a finite segment of time or, equivalently, via an ensemble of states (trajectory along the extent progression). Within this assumption, each point on the extent scale can represent a particular point in time along the evolution path shared across the members of the given ensemble.

Generalized fluxes can quantify dynamics of non-metabolic variables

By grouping individual metabolites into pools, the metabolic system might be modeled with a more coarse-grain network that is simpler to be parameterized and computationally analyzed. For example, metabolites connected with the remaining system via only one incoming and one outgoing flux can be unified into a single network node. Similarly to metabolites, this argument also applies to metabolic fluxes. For example, parallel fluxes connecting two metabolites can be pooled into one.

So far, the framework is unable to handle parameters characterizing the metabolic system that are not metabolite concentrations. In the clinical setting, it is frequently more convenient to measure macroscopic physiological parameters instead of metabolite concentrations. For example, pulse wave velocity (PWV) is easily measured with a cuff at the upper arm and leg. At the same time, measuring the related plasma concentrations of oxidized low-density lipid cholesterol (ox-LDL) [1, 2] requires substantial laboratory effort.

The better the linear correlation between the non-metabolic parameter, e.g PWV, and the related metabolite concentration, e.g ox-LDL, the more justified is an inclusion of an additional edge between the two characteristic nodes (here: the edge between PWV and ox-LDL) into the metabolic network and the description with a generalized flux between them. The slope of this regression enters the stoichiometric matrix as S_{ij}, where i is the index of the dependent node, e.g. ox-LDL and j is the index of the edge leading to the independent node, e.g. PWV.

$$\frac{dX_i}{d\xi} = \sum_j S_{ij} \frac{dX_j}{d\xi} \tag{4}$$

To note, the differential at the right side of the equation can be termed the generalized flux v_j. For the edge in the reverse direction, S_{ji} is the reciprocal value of the slope.

Once, we include non-metabolic nodes into the molecular network, it becomes possible to incorporate nodes quantifying disease progression into the same network. Diseases are characterized by development of syndromes that have measurable parameters. For example, endothelial dysfunctions progression can be quantified by the reactive hyperemia index (RHI) [3] that can be included as a node related to the total and low density lipid cholesterol plasma concentrations. In this framework, the reaction extent ξ, so far interpreted as a concentration change, can be generalized as a **state progress variable** ξ, for example the extent of disease progression measured by quantifying a syndrome.

Non-stoichiometric fluxes are not new to MFA. A metabolic system exchanges matter and information with the environment. Each such exchange process can be represented as a flux, referred to as an exchange flux, which quantifies the rate of incoming or outgoing matter or information. Systemic incoming and outgoing exchange fluxes are not subject to mass conservation laws as regular fluxes, as they represent links to unlimited resources. Therefore, together with stoichiometrically-constrained metabolic fluxes, they form an extended stoichiometry matrix. In a formally similar manner, our definition of the extended stoichiometry matrix components and fluxes connects quantifiable non-stoichiometric variables as exchange fluxes.

The optimization problem in the GMFA framework

The vector of observables X of the metabolic system at a given state represents the metabolite concentrations and physiological variables measurement taken at that state. The difference between the observations at two states $\Delta X = X^B - X^A$ characterizes the progression extent ξ. The best fit set of generalized fluxes v satisfying equation 2 is obtained minimizing the squared difference between the observed state variable change ΔX and the best fit estimate of it Sv, the expected change of the state variable given the generalized fluxes v.

The best fit solution for fluxes might result in a deviation from the observed state variables due to conflicting experimental measurement errors as well as due to approximately inferred components of the extended stoichiometric matrix. Therefore, we allow the flux solution to result in a deviation from the observed state variables within an empirically set ϵ vicinity.

$$\begin{cases} v = argmin(v^T S^T S v - S^T \Delta X) \\ Sv \leq (1+\epsilon)\Delta X \\ Sv > (1-\epsilon)\Delta X \\ v \leq v_{max} \\ v > v_{min} \\ \epsilon = 0.2 \end{cases} \tag{5}$$

The solution is obtained with the Goldfarb-Idnani quadratic programming procedure [4], using the Quadprog v.0.1.8 software library [5]. The calculated generalized metabolic fluxes v are the best fit solution of equations 5.

The sequence of the ensemble members that corresponds to the minimal cumulative extent difference represents points on the shortest path (the smallest change of fluxes) between the metabolic states. Such a sequence can be obtained by ordering the organisms/patients by their proximity to a particular state, using a simplified metric in the flux space. Here, we define the metric as a distance between the flux profiles, using the following formula:

$$d(v^A, v^B) = \sum_{j=1}^{N} \begin{cases} 0 : v_j^A = 0, v_j^B = 0 \\ \frac{|v_j^A - v_j^B|}{max(|v_j^A|,|v_j^B|)} : max(|v_j^A|, |v_j^B|) \neq 0, sign(v_j^A) = sign(v_j^B) \\ 1 + \frac{|v_j^A - v_j^B|}{max(|v_j^A|,|v_j^B|)} : max(|v_j^A|, |v_j^B|) \neq 0, sign(v_j^A) \neq sign(v_j^B) \end{cases} \tag{6}$$

The metric is defined to measure the distance between states A and B. The metric is additive with respect to its components derived from individual fluxes. For each individual flux, the respective metric component is zero (minimal), when the flux value at both states A and B is zero or identical. The metric component equals one (maximal) when signs of the flux value at state A does not match that at state B. When the signs match, the metric reflects the relative difference between the flux magnitudes. In contrast to distance definitions, like the Euclidean one, our metric is normalized; thus the influence of large fluxes is lowered, compared to small fluxes that might change in sign.

Thus, following the metric, states A and B are considered identical when all flux magnitudes and signs match. The difference between A and B is maximal (equal the total number of fluxes, N) when all fluxes have different signs. Overall, the metric reflects the weighted number of flux perturbations observed in the system upon transition between two given states.

Appendix B. An example of extending the stoichiometry matrix with non-stoichiometric physiological variables

Kinetic models

A long-term study of patients with rheumatoid arthritis provided information on ox-LDL and PWV changes as a result of 6 weeks treatment with a daily 20

mg simvastatin and 10 mg ezetimibe [1]. The study reports a decrease of PWV by 0.71 m/s and ox-LDL by 19.7 U/L as a result of simvastatin treatment. With this initial information, we can use Equation 4 to estimate the coefficients S_{ik} and S_{jk}, where i and j denote the indices of PWV and ox-LDL, respectively, k denotes the index of the flux v_k from ox-LDL to PWV and points A and B are taken as the start and the end of the treatment period. We estimate the average change per week of treatment in both parameters as $\Delta_{AB}x_i = -0.118$ and $\Delta_{AB}x_j = -3.283$. Assuming that v_k is an exchange flux, we can set $S_{jk} = 1$ to obtain $S_{ik} = 0.006$ per week of treatment (i.e. per unit of the extent variable). Importantly, when ezetimibe is used as a treatment, applying the same method would provide a different value for the coefficient, i.e $S_{ik} = 0.02$. This is not surprising, since the estimates depend on the choice of the extent variables ξ . Indeed, two drugs have different mechanisms of action: simvastatin inhibits endogenous cholesterol production, while ezetimibe inhibits absorption of cholesterol in the small intestine. Each mechanism results in a different direction and extent magnitude of metabolic changes.

In an independent study of diabetes type 2 patients the authors estimated the effect of a 4-weeks long atrovastatin treatment on ox-LDL [6]. The value re-scaled per week is estimated as $\Delta x_i = -3.372$, which is close to the value of -3.283 obtained in the equivalent dose simvastatin study [1].

In a separate study [7], effects of atrovastatin therapy on PWV were evaluated in patients with hypertension and hypercholesterolemia in the duration of 26 weeks. The reported change in PWV re-scaled per week produces $\Delta x_i = 0.065$, which is lower than the value obtained in the simvastatin study (-0.118). The difference may be due to different basal level of arterial stiffness in patients with hypertension and hypercholesterolemia [7] compared to the patients with rheumatoid arthritis [1].

Appendix C. Supplementary File

For value codes of the *ethnicity* and the *retinopathy* variables, see the description of the NHANES tables *ridreth1* and *diq080*, respectively.

Supplementary Tables

Supplementary Table 1

Table 1: **Literature-based values in the states of non-diabetic (state A) and late diabetic (state B states of diabetes progression.** These values were used as inputs to compute the optimal fluxes as the best fit solution to Equation 5

**Metabolite / phys. variable**	**Non-diabetic**	**Diabetic**	**Maximal**
TG	0.2	1.2	6
HDL	1.1	1.5	4
VLDL	0.1	0.5	0.8
LDL	1.1	3	6
Ox-LDL	0.1	50	200
Pulse-wave-velocity	2	8.3	30
RHI	0.01	0.66	3
CIMT-avg-L	0.2	0.62	2
CIMT-avg-R	0.2	0.62	2
ROM	1	200	1000
BAP	1	2000	5000
hsCRP	0	1.8	10
FFA	0.7	1.2	1.5
Fat-lipids	0.5	2.1	2.52
Liver-FA	0.2	0.61	0.732
Liver-cholesterol	0.2	0.459	0.551
Protein	0.9	1.09	1.308
Glucose	3.8	6	7.2
HbA1C	1	5	20
Bilirubin	0.08	0.8	0.96
Creat	0.002	0.07	0.1
Ur-LpH	4	6	9
Ur-prot	0	5	20
Ur-Glc	0	0.5	10
Ur-ket	0	0.2	10
BMI	10	18	40
Haptoglobin	3	200	1000
Hb	1	20	100
Ferritin	1	300	2000
Erythrocytes	1	50	100
Endothelium	1	50	100
NO	23	24	25
Haematocrit	1	40	90
Bicarbonate	10	23	40
Iron	0	0.08	0.5
Ferritin-Iron	1	300	2000
ESR	0	5	60
Liver	0	1	100
ALT	0	35	100

Table 2: **Flux constraints matrix of the metabolic and physiological pathways driving diabetes complications.** It was compiled based on available database and literature information. Along with the stoichiometry matrix (Table 2), the flux constraints matrix is an essential input of the optimization procedure to compute the best fit fluxes through the system, using Equation 5.

**Flux**	v_{min}	v_{max}
RHI↔CIMT-avg-R	-∞	∞
RHI↔CIMT-avg-L	-∞	∞
NO→RHI	0	∞
Erythrocytes↔NO	-∞	∞
Endothelium↔NO	-∞	∞
Erythrocytes→Haematocrit	0	∞
Ex:Respiration→Erythrocytes	0	∞
Ex:CIMT-avg-L→Atherosclerosis	0	∞
Ox-LDL→CIMT-avg-R	0	∞
Ox-LDL→CIMT-avg-L	0	∞
HDL+VLDL→LDL	0	∞
LDL+ROM→ox-LDL	0	∞
ROM→hsCRP	0	∞
Ex:∞lammation→ESR	0	∞
Ex:∞lammation→hsCRP	0	∞
Ex:∞lammation→Ferritin	0	∞
Ex: Haptoglobin+Hb→Spleen	0	∞
Ex:Respiration→Bicarbonate	0	∞
Ex:Respiration→Haematocrit	0	∞
Ex:hsCRP+ox-LDL→Atherosclerosis	0	∞
Ex:ROM+BAP→Deactivation	0	∞
Ex:Food→FFA	0	∞
Ex:Food→LDL	0	∞
Ex:Food→Protein	0	∞
Ex:Food→Glucose	0	∞
Ex:Bilirubin→Bile	0	∞
Fat-lipids→HDL	0	∞
HDL→Liver-cholesterol	0	∞
Liver-cholesterol→Bilirubin	0	∞
FFA ↔Liver-FA	-∞	∞
FFA ↔Fat-lipids	-∞	∞
Liver-FA→Glucose	0	∞
Fat-lipids↔TG	-∞	∞
Fat-lipids→BMI	0	∞
Liver-FA→VLDL	0	∞
Protein→Ur-pH	0	∞
Glucose→Ur-pH	0	∞
Glucose→HbA1C	0	∞
HbA1C→Bilirubin	0	∞
FFA→Ur-pH	0	∞
Protein→Ur-prot	0	∞
BMI→Ur-prot	0	∞
Glucose→Ur-Glc	0	∞
Liver-FA→Ur-ket	0	∞
BMI→Creat	0	∞
Liver→ALT	0	∞
Iron → ROM	0	∞
Ferritin+Iron ↔Ferritin-Iron	-∞	∞
Haematocrit ↔Hb	-∞	∞
Hb→Iron	0	∞
Hb+ROM→HbA1C	0	∞
Hb→Bilirubin	0	∞

References

[1] K.M. Mäki-Petäjä, A.D. Booth, F.C. Hall, S.M.L. Wallace, J. Brown, C.M. McEniery, I.B. Wilkinson, Journal of the American College of Cardiology **50**(9), 852 (2007). URL http://www.sciencedirect.com/science/article/pii/S0735109707018293

[2] N.W. Tsai, L.H. Lee, C.R. Huang, W.N. Chang, Y.T. Chang, Y.J. Su, Y.F. Chiang, H.C. Wang, B.C. Cheng, W.C. Lin, C.T. Kung, C.M. Su, Y.J. Lin, C.H. Lu, Critical care (London, England) **18**(1), R16 (2014). URL https://pubmed.ncbi.nlm.nih.gov/24423248

[3] B.K. Koo, W.Y. Chung, M.K. Moon, Cardiovascular Diabetology **19**(1), 82 (2020). URL https://doi.org/10.1186/s12933-020-01062-z

[4] D. Goldfarb, A. Idnani, Mathematical Programming **27**(1), 1 (1983). URL https://doi.org/10.1007/BF02591962

[5] Quadprog: Quadratic programming solver (python). URL https://github.com/quadprog/quadprog

[6] A. Akalin, G. Temiz, N. Akcar, B. Sensoy, Endocrine journal **55**, 861 (2008)

[7] A.I. Kanaki, P.A. Sarafidis, P.I. Georgianos, K. Kanavos, I.M. Tziolas, P.E. Zebekakis, A.N. Lasaridis, American journal of hypertension **26**, 608 (2013)
