# Supplementary material for: Generalized metabolic flux analysis framework provides mechanism-based predictions of ophthalmic complications in type 2 diabetes patients
Source: Health Inf Sci Syst. 2023 Mar 29;11(1):18. doi: 10.1007/s13755-023-00218-x (PMC10060506; doi:10.1007/s13755-023-00218-x)
Supplement: Supplementary file 2 — Supplementary file2 (PDF 94 kb) [file 13755_2023_218_MOESM2_ESM.pdf]

id	gender	ethnicity	age	dm_years	diabetes	retinopathy	BMI	Hb	Haematocrit	HbA1c	LDL	HDL	Cholesterol	TG	Glucose	age_group	SBP	dm_group
31132	1	3	70	7	1	2	24.74	14.5	42.6	6.9	1.94	1.53	3.80	0.73	8.72	70-80	130	<25
31162	2	3	71	31	1	1	29.6	12.7	36.3	8	3.672	1.22	5.79	1.97	6.89	70-80	100	>=25
31519	2	3	57	17	1	2	46.24	14.6	42.9	8	1.94	1.68	4.55	2.06	11.89	<60	128	<25
31559	2	3	66	8	1	2	30.52	15.7	47.5	6	2.276	1.32	5.15	3.40	7.39	60-70	138	<25
31619	2	3	31	11	1	2	30.72	12.8	37	7	3.414	1.97	5.87	1.07	11.17	<60	124	<25
31945	1	3	67	4	1	1	29.33	15.9	46.9	5.1	3.414	1.11	5.56	2.27	6.61	60-70	104	<25
32035	1	4	60	15	1	2	26.85	13.4	38.8	9	2.56	1.14	4.22	1.14	5.00	60-70	134	<25
32063	1	3	12	4	1	2	18.26	15.1	44.4	9	2.379	3.15	5.79	0.55	21.34	<60	104	<25
32294	2	3	77	15	1	1	26.79	13.9	41.8	7	1.526	1.6	3.75	1.36	9.22	70-80	140	<25
32443	2	3	69	1	1	2	42.08	13.5	42.8	6	1.603	1.6	4.29	2.37	8.17	60-70	170	<25
32585	2	4	16	4	1	2	25.56	14	39.8	10	2.56	2.28	5.02	0.42	7.33	<60	110	<25
32601	2	3	58	16	1	1	35.34	17.4	51.3	7.1	2.431	1.55	4.53	1.21	9.06	<60	102	<25
32791	2	3	28	3	1	2	27.14	14.7	43.6	11	3.62	2.2	7.78	4.27	11.72	<60	106	<25
32880	2	4	62	7	1	2	32.2	11.6	34.5	7	2.121	1.42	4.01	1.01	9.83	60-70	153	<25
32918	2	4	64	25	1	2	34.62	12.1	36.8	6.8	2.353	1.53	4.45	1.24	7.61	60-70	138	>=25
33077	1	3	72	3	1	2	29.97	15.7	44.9	6.1	1.422	1.19	3.52	2.00	7.28	70-80	130	<25
33236	2	3	68	3	1	2	30	11.9	34.8	6	2.974	1.47	4.94	1.05	7.17	60-70	120	<25
33454	1	4	84	20	1	2	25.19	15.6	46.7	7	2.457	1.29	4.22	1.02	4.89	>=80	130	<25
33691	2	4	58	26	1	2	33.79	10.1	31	11	4.939	2.33	7.73	1.01	5.78	<60	172	>=25
34249	2	3	81	3	1	2	23.59	14.6	44.3	6	2.302	1.81	4.84	1.58	8.00	>=80	136	<25
34605	1	3	53	10	1	1	35.43	15.2	46.1	8	2.224	1.29	4.09	1.27	10.78	<60	127	<25
34769	2	3	70	7	1	1	26.95	15	44.3	8.1	1.965	1.63	3.98	0.85	10.28	70-80	152	<25
34965	2	1	59	14	1	2	21.83	14.3	41.3	12	3.957	1.37	5.90	1.24	16.78	<60	96	<25
35157	1	4	46	8	1	2	27.83	14.7	43.9	11	1.474	1.24	3.21	1.08	11.45	<60	152	<25
35198	2	5	61	8	1	2	23.67	12.9	37.7	6.7	2.741	1.86	4.94	0.75	8.95	60-70	137	<25
35232	2	1	67	1	1	2	27.56	13.9	39.5	7	3.284	1.37	5.92	2.79	7.06	60-70	144	<25
35675	2	3	70	15	1	2	29.52	13.1	39.2	6	2.586	1.24	4.99	2.54	7.00	70-80	150	<25
35884	1	3	60	17	1	1	42.46	14.9	44.1	7.6	1.836	1.11	3.72	1.67	8.56	60-70	186	<25
36119	1	2	83	1	1	2	29.24	16.9	50.5	11	2.767	1.01	4.78	2.20	7.39	>=80	200	<25
36184	2	3	59	4	1	2	31.3	12.2	36	6	3.336	1.19	5.74	2.63	6.00	<60	115	<25
36408	1	1	42	5	1	1	46.04	12.4	37	8	0.569	0.75	2.79	3.22	2.50	<60	90	<25
36649	2	1	69	4	1	2	32.21	15.1	46.8	6	2.379	1.84	4.58	0.78	6.33	60-70	128	<25
36896	2	4	60	12	1	2	25.56	12	36.5	7	3.414	1.89	5.79	1.10	6.89	60-70	126	<25
37050	1	4	69	12	1	2	29.04	15.5	48.2	12	1.474	0.96	2.79	0.80	8.17	60-70	138	<25
37187	1	4	44	1	1	2	25.68	14.2	41	6.5	2.379	1.4	4.65	1.93	6.39	<60	146	<25
37279	2	3	43	6	1	1	31.76	12.5	36.6	5	3.103	1.94	5.66	1.34	4.44	<60	120	<25
37373	2	3	77	17	1	2	33.26	13.3	39.6	7	1.034	2.33	3.96	1.28	9.56	70-80	140	<25
37436	1	3	77	55	1	1	43.79	12	34.3	6	2.121	0.59	3.52	1.74	8.17	70-80	136	>=25
37769	2	3	64	17	1	1	40.1	11.4	33.4	6.1	1.81	1.58	4.24	1.89	6.83	60-70	92	<25
37910	2	1	60	16	1	1	38.81	12.1	37.3	9	1.784	1.27	3.65	1.28	10.11	60-70	142	<25
38399	2	1	13	1	1	2	36.34	14.9	44.9	10	2.586	1.11	4.32	1.34	16.39	<60	104	<25
39051	2	3	77	20	1	2	27.64	15	43.6	5	3.103	1.4	5.12	1.36	8.22	70-80	126	<25
39433	2	3	80	15	1	2	32.94	13.1	39.2	7	2.56	1.22	4.37	1.29	13.22	>=80	182	<25
39463	2	3	50	25	1	1	23.05	13.2	38	9.9	1.784	1.6	4.03	1.40	21.45	<60	146	>=25
39615	1	3	46	8	1	2	39.26	14.9	42.8	9.1	1.603	1.11	3.00	0.60	7.17	<60	134	<25
40318	1	5	74	24	1	1	29.28	15.6	48	8	2.741	0.96	5.74	4.44	10.56	70-80	112	<25
40726	1	3	48	12	1	2	38.61	14.6	42.2	6	1.5	1.01	3.00	1.10	7.11	<60	114	<25
40838	2	3	73	11	1	2	24.74	13.4	39.7	7	3.724	1.86	6.08	1.08	10.17	70-80	176	<25
41094	1	3	46	2	1	2	42.67	15.6	46.6	5	2.379	0.93	4.16	1.88	6.44	<60	117	<25
41501	1	3	70	63	1	2	36.27	13.5	40.5	7	1.991	1.24	4.09	1.89	9.11	70-80	144	>=25
41542	2	4	60	13	1	2	32.83	12.5	38.3	5.5	2.457	1.63	4.78	1.55	10.50	60-70	126	<25
41613	1	4	71	16	1	2	32.42	14.9	43.8	6.2	2.534	1.01	4.03	1.06	8.00	70-80	116	<25
42154	1	3	75	7	1	2	25.31	16	46.1	5	0.879	0.83	2.61	2.00	12.56	70-80	168	<25
42238	1	3	30	21	1	2	22.78	14.3	41.7	7	2.431	1.29	4.06	0.75	12.56	<60	100	<25
42479	2	4	55	2	1	2	43.5	14	43.1	17	3.853	1.29	5.61	1.04	6.22	<60	144	<25
42484	2	3	72	32	1	1	42.44	11.2	33.3	7	1.448	1.68	3.65	1.13	3.94	70-80	134	>=25
42721	1	3	18	6	1	2	22.36	15	44.2	14	2.121	1.81	4.99	2.33	17.33	<60	102	<25
42745	2	1	51	6	1	2	40.53	12.2	36.6	7	2.095	1.5	4.16	1.23	6.89	<60	118	<25
42751	1	3	76	23	1	2	34.52	12.4	35.2	5.6	2.327	1.11	4.32	1.90	7.72	70-80	149	<25
42850	1	3	53	13	1	1	25.65	16.5	47.3	6	2.56	1.09	4.11	1.03	9.11	<60	116	<25
42900	1	4	63	26	1	2	36.77	15.5	46.7	6	3.646	1.09	5.46	1.60	7.11	60-70	148	>=25
42901	1	4	33	11	1	1	44.23	14.3	42.5	9	3.62	0.83	5.74	2.80	17.89	<60	134	<25
42948	1	3	41	3	1	2	32.93	15.9	45.9	6.7	2.327	0.98	4.29	2.14	8.28	<60	120	<25
43227	1	4	39	4	1	2	39.16	17.2	50.9	7	2.327	1.06	3.67	0.62	5.50	<60	126	<25
43415	1	3	52	7	1	2	34.21	15	43.7	10	2.276	1.27	4.01	1.03	15.83	<60	136	<25
43529	2	3	38	1	1	2	37.7	14.4	42.7	5	4.112	1.42	5.77	0.49	6.89	<60	128	<25
43558	1	3	69	24	1	1	24.15	15.9	45.4	5	1.81	1.5	3.57	0.58	6.83	60-70	180	<25
43573	1	3	77	17	1	2	26.26	14	41.3	8	2.121	1.11	4.27	2.24	7.06	70-80	132	<25
43656	1	4	80	7	1	2	27.07	11.4	33.7	8	1.629	0.93	3.08	1.12	5.50	>=80	136	<25
43710	1	3	50	5	1	2	38.22	15.2	42.8	6	2.353	1.03	4.40	2.18	9.56	<60	124	<25
43816	2	3	77	12	1	1	36	13.5	38.5	7	1.526	1.16	3.28	1.32	9.67	70-80	118	<25
43995	2	3	80	15	1	1	30.54	11.8	35.2	7.6	2.095	1.45	4.16	1.33	4.83	>=80	174	<25
44129	2	2	51	15	1	2	26.19	15.8	45.9	5	3.776	1.11	5.64	1.65	17.50	<60	146	<25
44291	2	3	39	21	1	2	23.02	12.5	37.2	3	6.155	0.91	7.96	2.00	5.94	<60	112	<25
44296	2	3	73	7	1	2	33.43	12.6	37.2	6	2.172	1.81	4.42	0.95	7.39	70-80	136	<25
44433	1	3	66	13	1	2	29.39	16	47.2	7	2.069	0.98	3.59	1.18	8.17	60-70	110	<25
44474	2	2	73	5	1	1	26.46	12	35.2	6	3.026	1.45	5.30	1.82	7.89	70-80	126	<25
44517	1	3	61	6	1	2	32.31	14.6	41.4	8	2.948	1.24	4.53	0.71	12.28	60-70	102	<25
44578	1	3	53	25	1	2	26.13	15.2	44.9	8	2.56	2.2	5.09	0.72	13.11	<60	122	>=25
44633	1	3	60	19	1	2	27.49	15.5	44.5	11	1.371	1.4	3.26	1.07	5.67	60-70	132	<25
44970	2	3	56	11	1	2	48.94	10.9	31.5	5	2.896	1.01	4.55	1.40	5.56	<60	108	<25
45028	1	3	61	6	1	2	34.08	14.3	40.7	7	1.371	0.83	2.90	1.51	6.67	60-70	128	<25
45311	1	4	68	13	1	2	35.34	12.2	36.6	5	2.793	1.16	4.42	1.02	8.33	60-70	140	<25
45433	2	3	49	28	1	1	42.85	14.5	42.6	10	1.733	1.42	4.01	1.86	8.00	<60	154	>=25
45491	1	3	78	30	1	1	26.06	14.1	41.6	8	2.276	1.11	4.24	1.86	9.45	70-80	146	>=25
45625	1	2	76	11	1	1	28.38	14.4	43.4	7	3.31	1.45	5.74	2.16	5.50	70-80	144	<25
45788	2	2	64	33	1	2	41.05	14.4	41	6	2.922	1.66	4.94	0.81	10.33	60-70	124	>=25
45815	1	3	67	25	1	1	24.29	13.8	40.2	7.9	1.552	1.22	3.70	2.02	8.45	60-70	134	>=25
45829	1	3	80	5	1	2	20.22	11.5	32.5	6	2.224	0.93	3.70	1.18	5.50	>=80	196	<25
45894	1	3	60	12	1	2	29.92	15.1	43.8	8	2.25	1.73	5.07	2.38	19.39	60-70	150	<25
46221	1	3	55	7	1	1	37.14	15.2	44	7	3.595	0.75	5.97	3.58	21.61	<60	118	<25
46252	2	3	80	16	1	2	24.01	13.5	40	6	3.776	1.81	5.95	0.77	6.78	>=80	134	<25
46315	2	3	76	11	1	2	32.55	13.5	38.1	6	1.629	1.5	4.09	2.11	6.95	70-80	186	<25
46320	2	4	45	10	1	2	31.33	12.7	39.3	9	3.414	1.68	5.56	1.03	9.89	<60	124	<25
46343	2	4	65	23	1	2	42.52	10.7	33.7	6.7	3	1.29	5.97	3.66	12.33	60-70	132	<25
46348	1	4	58	8	1	1	40.6	14.3	42.8	6	3.258	1.55	5.87	2.32	14.50	<60	140	<25
46470	1	3	66	3	1	1	29.06	14.1	42.3	5	1.707	1.16	3.70	1.79	7.33	60-70	110	<25
46477	1	3	68	22	1	1	38.18	11.9	34.5	7	1.371	1.47	3.10	0.57	5.22	60-70	124	<25
46491	1	3	78	23	1	2	23.38	12.9	38.3	6	1.603	1.09	2.95	0.54	5.33	70-80	124	<25
46493	2	4	66	4	1	1	29.86	11.8	35	8	3.672	2.02	6.05	0.79	9.72	60-70	132	<25
46704	1	4	59	2	1	2	35.16	15.6	45.7	6	4.422	1.29	6.31	1.31	7.06	<60	118	<25
46807	1	3	66	5	1	1	26.72	14.1	42.2	12	1.319	0.65	3.15	2.60	15.89	60-70	114	<25
46832	1	3	19	10	1	2	26.39	15.5	47.1	8	2.25	1.27	4.03	1.12	15.06	<60	114	<25
47010	2	2	62	38	1	1	32.41	13.2	37.4	18	1.862	1.42	4.03	1.64	21.50	60-70	138	>=25

47073	1	3	71	8	1	2	37.8	14	41.2	6	3.233	0.91	5.07	2.01	8.67	70-80	118	<25
47304	2	3	56	52	1	2	32.8	14.5	41.7	7	2.56	0.85	4.09	1.49	8.50	<60	116	>=25
47397	1	3	62	20	1	2	39.95	14.7	42.2	6	0.957	1.19	2.64	1.07	6.17	60-70	134	<25
47594	1	3	80	15	1	1	30.48	10.8	32.9	6	1.965	1.22	3.54	0.81	7.17	>=80	138	<25
47778	2	3	67	8	1	2	31.54	10.8	31.8	8	1.836	1.27	3.44	0.72	5.89	60-70	104	<25
47825	2	2	69	19	1	2	21.97	12.5	37.5	5	3.181	1.4	5.33	1.66	5.17	60-70	168	<25
48133	1	3	52	1	1	2	36.26	17	49.1	6	1.784	0.83	3.10	1.05	7.95	<60	136	<25
48153	2	3	80	28	1	2	23.68	11	32.8	5	2.146	1.11	3.83	1.27	7.17	>=80	126	>=25
48433	1	3	74	14	1	2	32.18	13.9	40.2	8	2.25	1.22	4.16	1.51	7.72	70-80	124	<25
48820	1	3	78	23	1	2	30.66	12.6	37.6	8	3.724	1.5	6.62	3.05	12.78	70-80	139	<25
49170	2	3	62	6	1	2	37.17	15.1	42.6	5	1.655	1.27	3.70	1.72	7.28	60-70	98	<25
49177	2	1	63	1	1	2	43.14	12.6	37.3	2	1.086	0.83	3.54	3.58	5.39	60-70	114	<25
49416	1	3	61	9	1	2	36.37	16.3	46.7	7	2.379	1.01	5.07	3.70	7.78	60-70	158	<25
49459	1	4	33	5	1	2	28.63	14.4	42.4	5	3.414	1.32	5.07	0.71	6.89	<60	118	<25
49659	2	3	74	5	1	2	27.22	13.9	41.3	7.4	0.724	1.29	3.54	3.32	8.28	70-80	128	<25
49837	1	1	21	6	1	1	27.44	16.2	45.3	9	2.25	1.09	4.19	1.86	13.50	<60	94	<25
49980	1	3	13	4	1	2	39.47	15.2	45.2	6	2.896	0.96	4.73	1.94	5.39	<60	108	<25
50093	1	3	78	66	1	2	34.44	16.2	46.8	7.6	1.345	1.11	3.26	1.74	14.17	70-80	128	>=25
50108	1	3	51	13	1	2	32.88	14.5	42.1	9.1	2.819	1.32	4.81	1.46	12.83	<60	124	<25
50365	1	3	64	10	1	2	31.1	12.1	35.2	7.2	1.241	1.5	3.65	1.95	8.00	60-70	128	<25
50450	2	4	66	5	1	2	35.3	12.1	37.4	8	2.767	1.81	5.17	1.28	9.28	60-70	144	<25
50545	2	3	71	11	1	2	25.65	12.9	38.3	6	2.095	1.71	4.55	1.66	7.50	70-80	132	<25
50551	1	3	76	8	1	2	36.54	14.7	43	7.4	0.931	1.68	3.80	2.60	6.56	70-80	126	<25
50625	2	2	60	4	1	2	27.9	14	40	6	1.267	1.53	3.44	1.40	5.61	60-70	138	<25
50679	2	3	74	9	1	2	32.35	12	33.9	6.6	2.198	1.55	4.03	0.60	6.50	70-80	156	<25
51104	2	3	40	19	1	1	23.35	13.8	41.1	7	3.155	1.53	5.07	0.82	5.61	<60	102	<25
51140	2	4	60	3	1	2	37.6	13.3	39	6	2.638	2.17	5.12	0.69	7.89	60-70	130	<25
51190	2	4	68	55	1	2	30.25	14.8	44.8	6	2.095	1.19	4.06	1.70	6.11	60-70	108	>=25
51465	2	3	50	1	1	2	34.7	14	41.4	7	1.862	1.19	3.47	0.88	6.06	<60	138	<25
62228	1	3	50	2	1	2	43.4	134.9	41.1	6.6	2.896	0.85	4.73	2.17	11.33	<60	116	<25
62282	2	4	57	1	1	2	23.7	93.85	42	6	2.715	1.86	4.97	0.85	4.67	<60	108	<25
62317	1	3	46	13	1	2	27.6	128	42.4	7.1	3.414	1.53	5.69	1.65	5.44	<60	116	<25
62353	1	3	80	30	1	2	27.8	55.85	39.5	7.2	1.733	1.01	3.10	0.77	12.33	>=80	116	>=25
62366	1	3	80	5	1	2	21.7	63.8	33.8	5.5	1.836	1.14	3.36	0.85	7.50	>=80	180	<25
62413	1	3	65	11	1	2	40	7.45	43.8	5.8	1.086	1.63	3.59	1.91	3.89	60-70	136	<25
62686	2	3	80	5	1	2	38.7	101.8	40.1	5.8	3.388	1.34	5.56	1.83	8.17	>=80	159	<25
62727	2	3	80	15	1	2	30.8	79.1	35.2	6	3.103	1.68	5.46	1.47	7.33	>=80	217	<25
62861	2	3	80	7	1	2	18.4	64.1	44.3	7	2.922	1.97	5.20	0.69	8.72	>=80	140	<25
62945	2	4	48	10	1	2	28.1	52.15	32	6	2.302	1.22	4.06	1.21	10.00	<60	114	<25
62948	1	3	79	4	1	2	34.8	66.2	30.5	7	1.862	1.11	3.62	1.39	4.56	70-80	126	<25
62982	2	3	66	6	1	2	33.6	66.95	31.4	6.6	2.095	1.27	4.19	1.79	5.72	60-70	122	<25
63036	2	5	74	17	1	1	35.3	87	41.7	6	2.327	0.96	3.85	1.25	5.78	70-80	119	<25
63137	1	5	49	10	1	2	24.7	62.7	44.5	7.7	3.853	1.16	5.51	1.10	10.39	<60	132	<25
63211	2	3	46	4	1	2	40.8	142.2	36.2	6.5	2.431	1.37	4.84	2.24	7.78	<60	132	<25
63231	2	2	42	8	1	2	30.9	139.25	39.6	9	3.75	1.01	5.66	1.97	14.89	<60	122	<25
63248	1	3	75	30	1	1	26.6	80.15	41	7	2.689	1.22	4.65	1.66	8.78	70-80	134	>=25
63352	1	3	64	4	1	2	22.9	87.45	47.2	5	3.75	1.11	5.35	1.05	11.33	60-70	138	<25
63474	1	3	74	13	1	2	31.8	104.2	45	7.2	2.327	1.09	4.40	2.17	15.45	70-80	118	<25
63529	1	3	23	16	1	2	19.1	48.3	48.8	10.6	1.914	1.29	3.49	0.64	17.33	<60	116	<25
63579	1	3	63	54	1	1	24.3	40.9	29.4	8	1.655	1.81	3.83	0.78	10.06	60-70	140	>=25
63700	1	4	40	3	1	2	31.9	51.9	48	13	3.233	1.11	4.71	0.77	3.94	<60	146	<25
63739	2	4	71	26	1	2	20.4	41.3	31.5	9.1	1.681	1.27	3.34	0.87	17.78	70-80	190	>=25
63762	1	3	23	12	1	2	28.3	70.25	42.4	9	1.733	1.71	3.80	0.78	9.50	<60	116	<25
63879	1	3	76	16	1	2	23.6	70.55	37.5	6.9	2.25	1.19	3.96	1.15	8.50	70-80	147	<25
63887	2	3	64	19	1	2	40.1	91.05	38.3	7.6	1.5	1.14	4.55	4.19	13.50	60-70	124	<25
63909	2	3	62	12	1	2	32.2	119.1	37.3	6.1	2.793	1.55	4.97	1.34	7.17	60-70	134	<25
63915	2	3	40	2	1	2	36.7	90.65	35.9	14	1.888	0.91	4.11	2.87	9.50	<60	108	<25
63992	1	3	44	4	1	2	40	234.2	35.9	6.8	2.146	0.93	4.32	2.72	9.00	<60	130	<25
64013	1	5	80	10	1	1	27.4	79.5	43.6	6.3	3.155	1.4	4.99	0.95	8.89	>=80	142	<25
64071	1	4	67	22	1	2	27.1	56.65	44.3	6	1.733	1.5	3.67	0.97	6.06	60-70	128	<25
64127	2	5	69	9	1	2	30.1	39.2	31	7	2.353	1.03	4.01	1.37	9.67	60-70	110	<25
64164	1	4	47	4	1	2	51.1	106.1	35.7	6	2.87	1.09	5.56	3.49	13.83	<60	130	<25
64228	1	3	44	11	1	2	37	131.95	44.1	8	3.155	1.03	6.13	4.21	14.33	<60	128	<25
64231	1	2	68	31	1	2	35.8	60.95	38.8	2.1	1.965	1.22	3.75	1.27	14.06	60-70	94	>=25
64343	1	3	63	13	1	2	32.4	121.7	41.4	8	2.069	1.16	4.14	1.97	5.56	60-70	148	<25
64459	2	3	55	37	1	1	43.6	45.05	37.8	8.1	1.836	1.27	3.52	0.88	9.22	<60	110	>=25
64497	1	3	60	42	1	2	32.7	140.95	47.2	12	7.499	1.32	10.29	3.21	6.39	60-70	144	>=25
64559	2	3	70	15	1	2	39.4	6.85	41.3	8	1.707	0.93	3.62	2.15	11.28	70-80	128	<25
64625	1	4	69	34	1	2	26	69.75	38.2	7.1	2.172	1.34	3.93	0.88	7.39	60-70	162	>=25
64857	2	3	54	5	1	2	47.7	5.75	34.7	12	3.336	1.03	5.20	1.81	4.89	<60	128	<25
65014	2	3	59	3	1	2	33.7	100.5	41.3	7	3.801	0.88	5.95	2.78	6.83	<60	137	<25
65061	1	5	38	6	1	2	29.7	128.15	43.2	5.9	2.302	1.03	3.96	1.34	6.39	<60	128	<25
65163	1	3	70	11	1	2	31.3	69	43.1	6.7	1.914	1.14	3.54	1.08	8.72	70-80	132	<25
65289	2	4	73	25	1	2	29.3	6.7	38.7	7.1	3.646	1.24	5.53	1.41	7.28	70-80	158	>=25
65334	2	2	60	38	1	1	34.4	104.55	37.5	7	2.121	1.19	4.37	2.32	6.17	60-70	172	>=25
65395	1	5	77	16	1	2	32.8	67.6	41.8	36	2.379	1.16	4.24	1.50	7.45	70-80	122	<25
65505	1	5	55	13	1	2	22.4	75.4	43.2	6	1.241	1.53	3.59	1.81	8.00	<60	106	<25
65625	1	5	23	8	1	2	38.4	7.8	44.3	9.1	1.629	1.14	3.83	2.32	13.50	<60	114	<25
65711	1	5	46	12	1	2	27.6	66.15	40.4	8	3.672	1.19	5.33	1.04	9.11	<60	114	<25
65751	1	1	54	6	1	2	26.2	144.6	44.9	6.5	3.103	1.06	5.59	3.11	6.83	<60	128	<25
65838	1	2	63	4	1	2	30.7	121.5	41.2	6.8	3.776	1.27	5.97	2.05	7.45	60-70	142	<25
65876	1	4	36	3	1	2	46	64.15	44	13	1.164	1.4	3.34	1.67	5.56	<60	134	<25
65911	2	3	76	4	1	1	35.1	68.5	41.9	23	2.379	0.96	4.45	2.43	13.17	70-80	142	<25
66068	1	5	76	11	1	1	21.2	50.5	38.3	7	3.026	1.45	5.28	1.77	7.67	70-80	151	<25
66311	1	5	39	8	1	1	23	55.35	46.8	8	2.353	1.09	3.96	1.11	7.39	<60	96	<25
66504	1	3	70	5	1	2	36.1	113.65	47.4	8	1.5	1.45	3.80	1.84	11.17	70-80	146	<25
66563	2	4	73	1	1	2	34.6	63.05	38.6	6	2.741	1.97	4.99	0.61	6.06	70-80	142	<25
66712	1	5	31	4	1	2	25.9	51.45	39.5	6.7	1.965	0.91	3.49	1.34	6.95	<60	112	<25
66759	2	4	57	1	1	2	40.2	100.55	36.8	12	3.491	2.02	6.08	1.27	8.17	<60	142	<25
66982	2	3	73	38	1	2	17.8	39.1	29.6	8	1.965	1.16	3.67	1.16	6.67	70-80	166	>=25
67226	2	4	43	13	1	2	26.1	46.45	36.1	9	2.845	2.07	5.12	0.44	13.11	<60	116	<25
67339	1	3	70	9	1	2	25.9	69.3	46.7	6	2.896	1.06	4.42	1.02	7.00	70-80	120	<25
67348	1	5	16	1	1	2	26.3	101.6	43.6	6.5	4.086	0.96	6.08	2.25	7.95	<60	116	<25
67501	2	5	62	16	1	2	31.8	108.55	36.1	7.5	1.422	0.72	3.98	4.03	9.83	60-70	112	<25
67635	1	1	65	10	1	2	26.4	73.45	46	6.7	2.534	1.4	4.27	0.71	6.50	60-70	136	<25
67652	1	3	46	29	1	2	26.9	105.4	47.2	7.1	2.172	0.98	4.45	2.81	9.45	<60	128	>=25
67696	2	5	67	2	1	2	23.5	104.05	39.4	7.4	3.414	1.37	5.46	1.45	4.72	60-70	130	<25
67766	2	3	62	7	1	2	34.3	157.3	39.5	5.8	2.483	2.17	5.02	0.79	5.61	60-70	152	<25
67978	2	3	60	12	1	2	24.6	56.25	42.9	9	2.638	0.98	4.03	0.92	6.11	60-70	124	<25
68064	2	1	80	30	1	2	39.3	111.55	35.1	5	2.715	1.27	4.60	1.37	15.00	>=80	104	>=25
68330	2	3	69	53	1	2	29.6	94.25	39.8	6.5	1.758	1.16	3.47	1.16	8.89	60-70	124	>=25
68495	2	3	78	2	1	2	33.3	85.2	36.3	7.1	3.491	1.19	5.77	2.36	7.22	70-80	136	<25
68571	1	5	53	9	1	2	25.3	79	45.3	7	3.595	1.34	5.59	1.39	6.22	<60	114	<25
68582	2	4	42	5	1	2	32	48	44.3	11	5.534	1.11	7.19	1.16	18.67	<60	110	<25
68626	2	3	70	53	1	2	32.6	97.45	34.2	6.2	2.224	1.84	4.65	1.29	5.11	70-80	132	>=25

68699	2	4	18	2	1	2	24.5	46.65	42.2	11	3.905	1.5	5.74	0.73	8.95	<60	130	<25
68787	1	3	65	15	1	2	27.5	58.35	44.3	6.2	1.94	1.27	3.96	1.62	7.00	60-70	106	<25
68818	1	4	62	6	1	2	34.9	46.65	44.3	7	1.603	1.37	3.23	0.55	8.50	60-70	100	<25
68873	2	5	24	8	1	1	47.5	116.65	36.2	6.8	3.233	0.88	5.48	3.02	7.11	<60	105	<25
68902	2	4	78	35	1	1	26.7	40.35	35.7	5	1.81	1.68	3.83	0.76	3.83	70-80	144	>=25
68947	2	4	65	25	1	1	24.7	42.95	38.2	7	2.87	1.53	4.63	0.50	9.78	60-70	144	>=25
69014	1	3	78	18	1	2	36.2	79.85	43.4	6.9	1.19	0.75	3.52	3.45	9.17	70-80	120	<25
69428	2	3	42	7	1	2	47	63.3	39	7.1	2.483	1.32	4.47	1.45	6.67	<60	106	<25
69431	2	4	79	11	1	2	22.3	65.9	40	6.5	2.327	1.66	4.55	1.23	6.06	70-80	176	<25
69622	1	3	58	7	1	2	51.5	105.8	45.3	7.4	3.595	0.75	5.09	1.64	7.11	<60	112	<25
69854	1	5	65	15	1	2	41.4	106.15	42.1	10	2.56	1.01	4.68	2.44	7.22	60-70	128	<25
69884	1	5	56	3	1	1	27.6	61.8	43.7	8.3	1.293	0.85	2.79	1.44	6.95	<60	114	<25
69898	1	3	71	11	1	1	27.7	86	44.1	6	2.922	1.29	5.28	2.29	7.72	70-80	130	<25
70331	1	1	52	7	1	2	32.4	99.1	42.5	6.5	3.233	0.98	5.40	2.58	7.50	<60	116	<25
70368	1	4	55	19	1	1	42.5	45.25	30.2	8.5	2.327	0.91	3.78	1.19	8.83	<60	158	<25
70425	1	3	77	29	1	2	23.9	42.85	47	6	0.828	1.01	2.17	0.76	7.45	70-80	148	>=25
70443	2	2	52	15	1	2	36.9	59.05	38.8	10	2.405	0.78	3.59	0.89	6.78	<60	126	<25
70590	2	2	51	13	1	2	31.9	103.35	40.6	5.1	2.845	3.23	6.39	0.70	4.78	<60	134	<25
70718	1	3	75	5	1	2	30.2	59.6	39.5	6.5	2.715	0.93	4.09	0.98	6.39	70-80	114	<25
70785	2	4	41	4	1	2	57.1	60.6	34.8	6.7	1.603	1.22	3.23	0.88	8.17	<60	134	<25
71111	2	3	75	15	1	1	42.9	165.3	35.8	7	1.784	1.09	3.57	1.51	7.83	70-80	146	<25
71388	1	1	42	10	1	2	36.7	99.25	44.9	6	3.982	1.06	5.79	1.66	15.95	<60	192	<25
71438	2	4	70	53	1	1	34.6	120.4	34.8	6	3.207	1.73	5.92	2.17	6.83	70-80	142	>=25
71473	2	4	38	5	1	2	29.5	118.75	36.3	5	3.776	1.6	5.90	1.14	7.83	<60	122	<25
71522	2	3	63	12	1	1	32.8	121.65	40.8	7.1	5.069	0.96	7.21	2.61	11.56	60-70	142	<25
71527	2	3	54	11	1	2	37.1	105.3	35.4	9	3.051	1.09	4.86	1.57	7.11	<60	128	<25
71542	2	3	67	8	1	2	33.7	52.6	39.7	5.8	1.371	1.14	3.05	1.21	7.22	60-70	140	<25
71739	1	3	80	20	1	2	25.9	79.45	43.7	8	2.586	0.85	4.14	1.50	6.72	>=80	158	<25
71839	2	3	57	5	1	2	42.4	119.2	45.2	11.1	1.94	0.8	4.68	4.21	16.22	<60	116	<25
73559	1	3	72	15	1	2	28.9	17.2	24.9775	14	1.448	1.55	3.26	0.58	10.72	70-80	140	<25
73638	2	5	63	23	1	2	24.8	12.2	122.15	6.1	1.836	1.22	3.62	1.22	6.33	60-70	140	<25
73647	2	2	61	4	1	2	27.7	15	67.45	7.6	1.733	1.09	3.28	0.99	6.17	60-70	116	<25
73694	2	3	66	8	1	2	26.8	15.1	21.9645	7	2.172	1.37	4.94	3.05	7.39	60-70	112	<25
73706	1	3	54	2	1	2	30.8	16.2	134.5	5	1.836	1.06	3.85	2.11	10.22	<60	138	<25
73839	1	3	68	12	1	2	35.4	14.2	218.75	9.1	1.655	1.06	3.49	1.71	11.78	60-70	136	<25
74160	2	2	66	15	1	2	33.3	13.3	20.0555	5	2.25	1.37	4.40	1.72	6.00	60-70	102	<25
74205	1	5	65	7	1	1	22.2	14.6	21.3275	7	2.431	1.53	4.24	0.63	8.78	60-70	176	<25
74306	1	1	62	20	1	2	33.3	12.5	18.1055	7	1.138	1.06	3.52	2.90	18.22	60-70	120	<25
74374	2	5	70	24	1	2	22.6	12	18.6555	6.7	1.577	1.76	3.54	0.47	5.83	70-80	128	<25
74536	1	2	65	2	1	2	28.2	16	23.4555	6.7	1.888	1.32	4.40	2.58	6.95	60-70	112	<25
74588	2	3	74	19	1	2	30.2	14.1	20.8055	7	1.112	1.34	3.88	3.08	7.50	70-80	128	<25
74784	2	1	39	12	1	2	45.5	13.1	19.0055	7.5	4.034	1.55	6.98	3.07	9.17	<60	110	<25
74823	1	3	67	12	1	1	48.1	13.6	20.2055	8	2.379	1.27	4.34	1.55	9.72	60-70	118	<25
75127	1	4	59	19	1	2	30.1	12.1	17.8055	5	2.276	1.6	4.27	0.85	5.00	<60	106	<25
75146	1	4	49	14	1	2	43.1	13.9	20.5555	10	3.362	1.09	5.15	1.54	10.28	<60	185	<25
75384	1	3	61	14	1	2	29.2	16.2	23.1555	6.7	1.733	0.78	3.90	3.06	9.45	60-70	120	<25
75397	2	1	62	1	1	2	33.5	14.1	20.4555	5.6	2.896	1.68	5.40	1.80	6.28	60-70	140	<25
75467	2	3	39	20	1	2	25.8	14.4	20.9055	7	4.215	1.55	6.13	0.78	8.78	<60	106	<25
75495	1	4	66	6	1	2	43.1	14.1	21.5075	8	2.017	0.7	3.08	0.80	6.44	60-70	132	<25
75519	2	4	44	7	1	2	31.7	13.4	20.3055	8	2.017	2.02	4.29	0.59	6.83	<60	116	<25
75527	2	3	58	10	1	2	55.7	14	22.1555	7.2	1.629	1.42	3.59	1.16	10.22	<60	120	<25
75539	2	4	19	3	1	2	29.4	13.4	24.9	5	2.586	1.47	4.29	0.52	4.94	<60	112	<25
75573	2	1	67	20	1	2	38.4	12	17.9055	6.5	3.258	1.37	5.95	2.87	10.78	60-70	132	<25
75576	1	4	47	7	1	2	26.9	15	22.1575	6.7	3.129	1.19	4.76	0.94	21.17	<60	134	<25
75610	1	3	56	16	1	1	29.2	15.9	70.65	11	2.483	1.16	3.93	0.63	14.22	<60	120	<25
75625	2	4	60	18	1	1	42.4	13.1	20.0345	13	2.302	1.14	4.03	1.31	14.83	60-70	106	<25
75688	2	2	57	15	1	2	33.1	12	18.9205	10.1	3.077	1.27	4.94	1.28	7.78	<60	118	<25
75762	2	3	61	16	1	2	31.3	14.7	21.7055	7.2	1.526	1.24	3.59	1.80	7.95	60-70	122	<25
75789	1	3	58	2	1	2	27.5	14.5	21.0055	5.5	1.94	1.16	3.47	0.78	6.83	<60	114	<25
75978	1	3	71	26	1	2	38.2	14.4	21.0055	7.1	2.172	0.49	4.63	4.28	8.61	70-80	138	>=25
76033	2	2	69	29	1	2	33.7	10.8	16.4555	7.3	3	0.8	4.16	0.77	8.39	60-70	108	>=25
76110	1	3	22	14	1	2	17	13.8	135.95	7.9	1.267	0.91	2.90	1.59	18.39	<60	102	<25
76125	1	3	12	7	1	2	23.6	12.3	18.9055	7	1.94	1.81	3.93	0.41	11.11	<60	98	<25
76145	2	3	60	7	1	2	38	12.9	19.509	7.2	1.914	1.06	4.09	2.44	7.22	60-70	122	<25
76159	1	3	75	9	1	2	29.4	15	22.0055	7	1.965	1.42	4.63	2.71	11.95	70-80	134	<25
76192	2	3	66	10	1	2	32.5	12.7	18.9055	13	3.103	1.37	4.94	1.02	13.17	60-70	122	<25
76199	2	3	56	6	1	2	39.1	14.6	21.3555	6.2	3.103	1.16	5.72	3.15	6.67	<60	102	<25
76236	2	1	40	11	1	2	34.8	13.8	20.4555	4.7	2.431	1.63	4.65	1.32	6.17	<60	108	<25
76472	2	5	52	10	1	2	41.3	12.2	19.4055	7.5	2.276	1.5	4.76	2.14	7.28	<60	130	<25
76675	1	5	48	14	1	1	24	15.8	23.5555	7.4	3.233	0.93	5.04	1.93	8.50	<60	108	<25
76809	1	3	80	20	1	2	20.7	9.8	14.158	6	2.276	1.09	3.96	1.29	4.72	>=80	228	<25
76828	2	5	40	2	1	2	26.9	11.3	17.5585	6	3.827	1.01	5.40	1.24	4.89	<60	108	<25
76860	2	4	60	3	1	2	28.1	13.1	42.6	7	3.62	1.24	5.61	1.63	5.61	60-70	116	<25
76872	1	3	66	28	1	1	30.9	15.4	22.9555	13	4.344	1.19	6.54	2.23	12.11	60-70	120	>=25
76928	1	3	57	46	1	1	26.9	13.3	103.9	9	1.733	0.85	3.08	1.05	8.61	<60	96	>=25
77000	2	3	55	2	1	2	50.3	13.5	20.2945	6	2.276	1.16	3.93	1.07	11.11	<60	116	<25
77010	1	1	44	10	1	2	25.6	14.8	21.6905	9	2.948	1.42	4.91	1.16	15.72	<60	134	<25
77058	2	1	17	14	1	2	26.3	13.7	20.3555	8.1	1.629	1.58	3.44	0.51	14.50	<60	102	<25
77399	2	3	80	19	1	2	33.8	13.3	20.4055	7.2	2.043	1.68	4.37	1.39	11.61	>=80	124	<25
77443	1	3	64	5	1	2	33.1	13.7	29.15	5.9	2.922	1.22	4.94	1.73	7.17	60-70	116	<25
77481	2	1	54	19	1	2	41.3	13.8	20.5555	10	3.853	1.16	5.59	1.25	9.56	<60	126	<25
77548	1	3	69	20	1	2	27.9	14.7	21.1055	7.2	2.095	1.29	4.09	1.54	7.11	60-70	132	<25
77560	2	4	60	5	1	1	39.1	13.4	19.527	7.2	3	1.53	5.25	1.58	8.33	60-70	136	<25
77600	2	2	58	13	1	2	41.2	14.3	22.025	9	2.56	1.14	4.84	2.50	8.83	<60	130	<25
77682	1	4	50	10	1	1	30.5	14.6	22.0055	11	2.353	1.16	3.80	0.64	12.33	<60	126	<25
77717	1	5	48	13	1	1	38.2	12.1	18.0055	13	1.862	0.85	3.62	1.98	14.11	<60	136	<25
77815	1	3	66	5	1	1	30.7	13.7	19.6555	6.9	2.25	0.96	3.67	0.99	7.39	60-70	108	<25
77822	1	3	77	2	1	2	37	16.1	257.35	5.6	1.655	0.98	3.57	2.06	8.72	70-80	110	<25
77938	1	5	72	32	1	2	35.7	15.2	23.014	8.2	1.888	0.8	3.15	1.04	8.17	70-80	168	>=25
77999	1	2	51	7	1	2	22.6	13.8	42.15	9.2	3.336	1.78	5.33	0.47	9.28	<60	128	<25
78028	1	1	64	5	1	2	26.8	12.5	19.4485	6.8	2.121	0.72	3.47	1.33	7.22	60-70	116	<25
78138	1	3	66	3	1	2	25.3	16.2	23.0555	7	3.284	1.06	5.02	1.45	7.83	60-70	134	<25
78145	2	4	44	5	1	2	43.5	12.6	18.9055	8.5	2.146	1.73	4.11	0.52	7.39	<60	130	<25
78227	2	3	76	6	1	2	22.9	9.6	14.3575	7	3.129	1.66	6.08	2.80	11.78	70-80	161	<25
78497	1	4	64	7	1	2	24	14	31.9	6	2.327	1.42	4.01	0.57	11.61	60-70	152	<25
78665	1	4	48	13	1	2	26.1	15.1	75.95	11	5.069	1.45	7.01	1.06	7.56	<60	122	<25
78737	2	4	71	11	1	2	49.5	10.4	15.9795	6	2.922	1.06	4.58	1.28	5.61	70-80	163	<25
78747	2	3	70	19	1	2	26.3	13.8	20.3555	7.3	1.914	1.32	3.75	1.15	6.78	70-80	130	<25
78952	2	5	44	14	1	2	29.2	12.9	19.613	9	2.612	0.85	5.12	3.64	10.28	<60	114	<25
78968	2	3	63	6	1	2	25	14.8	21.8055	6.4	4.448	1.11	6.52	2.08	6.17	60-70	136	<25
78974	1	4	54	22	1	2	29.1	15	47.9	7	2.715	1.5	4.58	0.77	8.56	<60	110	<25
79016	1	3	80	7	1	2	27.9	14	20.4055	7.5	2.689	1.16	4.14	0.61	4.39	>=80	108	<25
79138	2	4	60	15	1	2	39.2	12.9	19.511	8.3	2.896	1.4	4.84	1.19	9.83	60-70	156	<25
79206	1	3	72	22	1	2	28.4	13.4	20.358	6.5	2.302	1.29	4.34	1.66	6.89	70-80	134	<25
79270	2	4	66	1	1	2	41.4	11.6	18.1075	7.5	1.474	1.68	3.44	0.62	6.17	60-70	132	<25

79340	2	3	31	7	1	2	28.9	11.4	18.1055	5.7	1.603	1.53	4.14	2.18	5.67	<60	116	<25
79437	1	4	60	31	1	1	51.8	14.1	21.009	8	2.664	1.03	3.93	0.51	4.00	60-70	106	>=25
79544	2	3	52	6	1	2	25.3	12.7	79.9	6.9	2.689	1.06	4.42	1.49	8.33	<60	124	<25
79557	2	3	73	13	1	2	31.2	13.9	20.8665	5.5	3.207	1.4	5.38	1.67	6.44	70-80	112	<25
79833	1	5	54	10	1	2	25.4	13.7	21.7555	6.5	1.784	1.09	4.37	3.28	8.67	<60	124	<25
79889	2	3	76	6	1	2	35.1	13.7	19.9495	6.3	2.224	1.6	4.81	2.16	9.56	70-80	128	<25
79967	2	3	52	22	1	2	45.4	14.1	21.5555	11.2	2.017	1.09	3.83	1.59	13.22	<60	123	<25
80179	2	3	75	3	1	2	26.6	15.4	21.914	6.7	4.81	1.66	6.98	1.12	7.72	70-80	110	<25
80225	2	1	40	19	1	1	23.4	12.4	18.9055	6.7	1.784	1.76	3.70	0.35	4.39	<60	166	<25
80244	2	3	35	26	1	1	30.6	12.3	18.5075	5.9	2.793	2.22	5.51	1.08	6.72	<60	104	>=25
80285	1	5	50	2	1	2	29.3	15.7	96.85	8	2.327	1.58	4.55	1.41	7.33	<60	100	<25
80493	1	3	52	7	1	1	34.1	16	32.75	12.4	3.75	1.27	5.79	1.70	16.11	<60	142	<25
80786	2	3	80	18	1	2	29.2	12.5	18.8765	5	1.526	1.97	3.96	1.03	7.89	>=80	172	<25
80890	2	3	65	15	1	2	28.1	13.5	20.2555	7.3	3.801	1.01	5.90	2.35	10.17	60-70	136	<25
80939	1	3	47	6	1	2	31.4	15.5	48.5	6.9	2.379	1.34	4.16	0.95	7.33	<60	114	<25
81197	2	4	47	22	1	2	27.5	14	21.6665	8	2.121	1.91	4.55	1.15	23.39	<60	122	<25
81353	1	3	76	3	1	2	29	13.6	19.7555	8	1.293	0.57	2.79	2.05	8.22	70-80	142	<25
81524	1	2	73	36	1	1	24.4	11.2	17.4055	8.1	1.758	0.91	3.36	1.51	3.94	70-80	158	>=25
81581	1	5	80	20	1	2	25	15.4	23.5055	6.9	1.991	0.93	3.62	1.53	8.67	>=80	124	<25
81603	2	3	76	9	1	2	27.7	13.9	20.9055	6.8	2.612	1.19	4.91	2.44	7.00	70-80	148	<25
81777	2	3	63	13	1	2	31.2	14	21.7055	10	2.896	1.47	4.63	0.59	8.61	60-70	106	<25
81831	2	5	72	20	1	1	47.2	12.7	18.765	5.7	4.267	1.06	6.08	1.63	6.06	70-80	122	<25
81847	2	3	38	5	1	2	25.7	10.3	65.35	5.8	1.758	1.03	4.24	3.19	11.95	<60	120	<25
81849	2	1	44	6	1	2	31.4	12	17.8555	6.3	3.853	1.68	6.21	1.45	7.17	<60	108	<25
81952	1	5	56	1	1	2	25.2	14.3	21.2555	7.5	1.965	1.19	3.93	1.70	8.95	<60	116	<25
81962	2	3	38	27	1	2	41.9	13.9	19.8595	8	2.689	1.34	4.73	1.50	9.11	<60	130	>=25
81978	2	3	63	12	1	2	29.5	13.7	43.65	5.8	3.439	1.03	5.38	1.97	5.50	60-70	116	<25
82019	1	4	62	7	1	2	27.7	14.9	22.1115	5	3	2.02	5.35	0.72	8.61	60-70	136	<25
82335	1	3	52	12	1	2	32.9	14.9	21.8055	7	2.483	0.85	4.06	1.56	9.61	<60	128	<25
82372	2	3	44	14	1	2	30.6	17.1	46.35	9	4.577	1.22	6.47	1.45	11.95	<60	110	<25
82446	1	3	66	2	1	2	32.7	17.3	24.1555	6	2.069	0.93	3.41	0.93	7.06	60-70	118	<25
82741	1	3	70	5	1	1	41.6	15.5	22.61	5.1	2.224	0.8	4.68	3.62	6.39	70-80	90	<25
82755	2	3	32	2	1	2	50.8	14.9	40.7	8.9	4.551	1.01	6.80	2.73	8.89	<60	141	<25
82802	1	3	76	30	1	1	20.9	13.3	19.6555	7	1.603	1.99	3.78	0.38	19.83	70-80	110	>=25
82874	2	3	59	15	1	2	51.3	14.9	21.3555	9	2.534	1.24	4.73	2.08	16.78	<60	134	<25
82990	1	1	66	9	1	2	26.6	13.9	20.1555	7	2.767	0.98	4.55	1.77	5.78	60-70	112	<25
83118	2	5	71	3	1	2	20.7	13.1	19.1555	5.9	1.603	2.15	4.09	0.76	5.33	70-80	126	<25
83170	2	1	68	21	1	2	32.4	13.2	60.05	7.1	1.94	1.47	3.98	1.25	10.72	60-70	138	<25
83203	2	3	61	4	1	1	27.9	15.1	73.55	6.4	3.233	1.42	5.46	1.74	11.33	60-70	115	<25
83244	1	3	80	79	1	2	20.9	12.7	18.6555	6	2.017	1.29	3.57	0.58	7.56	>=80	118	>=25
83321	2	4	80	20	1	2	27.9	12.6	18.725	7	1.629	2.2	4.16	0.76	3.72	>=80	158	<25
83424	2	4	57	1	1	2	27.6	12.8	18.9055	8	4.37	1.63	6.44	0.96	9.22	<60	116	<25
83426	2	3	56	18	1	2	36.5	14	21.2555	7.6	3.982	1.47	7.11	3.64	17.83	<60	150	<25
83437	2	4	49	5	1	1	38.9	10.5	16.2555	6.8	2.353	1.5	4.37	1.12	7.61	<60	166	<25
83507	1	2	50	7	1	1	27.5	15.3	71.1	12	3.491	1.24	5.64	1.98	16.50	<60	126	<25
83557	1	4	61	2	1	2	26.5	13.4	20.6555	6.5	1.836	1.45	3.54	0.58	7.11	60-70	134	<25
83755	1	4	67	9	1	2	28.8	14.8	21.9525	7.3	2.922	1.47	4.89	1.08	15.78	60-70	132	<25
83757	2	2	57	25	1	1	35.4	13.6	20.1555	12	4.06	1.11	5.87	1.55	22.11	<60	146	>=25
83764	1	3	14	3	1	2	18.8	14.6	20.8055	7.3	2.767	1.01	4.24	1.01	20.50	<60	106	<25
83849	1	2	71	14	1	2	27.6	7.2	67.2	8	1.19	0.91	2.30	0.45	4.22	70-80	132	<25
83886	1	3	74	6	1	2	27.2	13.1	19.6885	6	2.043	2.04	4.55	1.02	6.83	70-80	142	<25
83911	2	4	43	12	1	2	30.7	8.4	14.2055	5.6	2.017	1.63	3.85	0.47	18.39	<60	126	<25
83996	2	4	60	5	1	2	33.1	14.3	21.2555	7	3.233	1.22	4.91	1.04	19.72	60-70	134	<25
84055	2	5	64	14	1	2	20.8	13.4	20.3055	6.6	2.172	1.89	4.71	1.42	7.72	60-70	156	<25
84125	2	3	72	12	1	2	30.4	12.8	19.3055	7	2.043	1.53	4.65	2.40	9.56	70-80	140	<25
84173	1	1	52	2	1	2	49	14.3	22.2255	6.5	1.758	1.06	3.59	1.70	8.33	<60	120	<25
84178	1	3	80	20	1	2	29.4	14.7	143.45	8	1.474	0.91	2.90	1.13	6.95	>=80	116	<25
84226	1	4	64	7	1	1	25	15.3	121.3	6	1.733	0.98	3.23	1.12	6.33	60-70	130	<25
84374	2	4	74	1	1	2	33.7	13.6	20.5555	5.3	1.836	1.4	3.67	0.97	6.22	70-80	146	<25
84424	1	3	69	2	1	2	33.5	14.4	21.4055	8.3	2.689	1.06	4.63	1.90	23.84	60-70	150	<25
84495	2	3	53	3	1	2	29.5	14.7	22.4055	7.1	4.189	1.03	7.03	3.97	9.45	<60	106	<25
84534	2	1	62	22	1	1	24.8	11.1	30.05	3.5	2.638	1.24	4.27	0.87	2.78	60-70	158	<25
84575	1	4	61	16	1	2	38.8	15.4	95.85	7.3	2.405	1.16	4.11	1.21	6.11	60-70	168	<25
84582	1	4	66	16	1	1	37.6	9.6	15.1055	6.9	0.75	0.96	2.40	1.55	10.78	60-70	132	<25
84602	2	3	63	13	1	2	31.1	13.6	20.564	6.6	3.233	1.22	5.38	2.01	6.78	60-70	121	<25
84622	1	4	80	75	1	2	29.9	14.2	21.2555	7.2	1.784	2.04	4.03	0.43	8.50	>=80	116	>=25
84776	1	4	47	22	1	2	23.4	12.3	106.15	8.1	1.371	1.42	2.92	0.31	1.17	<60	124	<25
84805	2	4	53	13	1	2	33	13.6	51.45	7	3.801	1.01	5.69	1.91	14.78	<60	134	<25
84832	1	3	58	4	1	2	23	15.5	24.16	7	1.836	1.06	3.13	0.51	5.50	<60	116	<25
84885	2	4	61	10	1	2	36.7	12.5	19.5875	6.2	1.707	1.34	3.31	0.58	7.95	60-70	144	<25
84944	2	3	60	2	1	2	29.1	14.5	42.35	7.5	5.353	0.96	7.65	2.92	10.56	60-70	112	<25
85043	1	4	26	19	1	2	29.8	15.2	49.55	9.6	2.819	1.45	4.76	1.05	14.83	<60	106	<25
85170	2	4	80	20	1	2	26.3	12.1	18.8055	5.7	2.508	1.89	4.63	0.49	8.11	>=80	142	<25
85192	2	3	53	5	1	2	22.5	14.9	103.3	6.3	1.81	1.81	4.47	1.86	6.78	<60	140	<25
85377	2	4	60	1	1	2	35.7	13.7	22.075	6.2	3.879	2.04	6.41	1.10	5.94	60-70	144	<25
85406	1	1	71	12	1	2	27.2	14.9	74.4	6.5	1.655	1.37	3.59	1.27	5.83	70-80	118	<25
85491	1	3	62	8	1	2	35.9	17.9	130.85	6.9	4.5	1.14	6.98	2.96	11.22	60-70	133	<25
85670	1	4	45	3	1	2	38	14.2	21.8865	7	1.81	0.96	4.01	2.73	8.50	<60	126	<25
85760	1	3	58	8	1	2	29.9	16	25.518	6.5	2.948	0.83	5.15	2.97	11.06	<60	106	<25
85777	2	2	68	19	1	2	33.8	12	18.9555	8.4	1.94	1.42	3.83	0.99	11.28	60-70	116	<25
85875	1	3	71	6	1	2	34.6	16.2	24.0055	5.7	1.836	1.55	3.78	0.86	7.22	70-80	120	<25
85904	1	4	66	3	1	1	30.1	13.4	20.639	6.7	2.948	1.14	4.40	0.67	7.50	60-70	144	<25
85948	2	4	38	3	1	1	37.5	13.2	20.3055	6.8	3.103	1.66	5.17	0.88	4.83	<60	134	<25
85968	2	4	61	12	1	2	26.9	14.5	20.7055	7	2.689	1.32	4.63	1.37	13.28	60-70	124	<25
86005	2	2	55	15	1	2	38.3	15.4	23.4555	8.6	4.551	1.34	6.88	2.15	17.28	<60	140	<25
86215	1	3	63	20	1	1	28.5	14.9	22.5555	6.2	1.526	1.84	3.75	0.84	7.22	60-70	120	<25
86221	2	2	57	27	1	1	40.6	13.3	20.303	7	2.146	1.03	3.83	1.42	8.67	<60	108	>=25
86262	2	4	31	1	1	2	39.5	13.1	65.35	5.1	2.224	1.27	3.85	0.81	6.17	<60	110	<25
86273	2	4	73	42	1	1	30.5	12.4	18.7555	7.7	2.121	1.55	4.22	1.18	8.45	70-80	128	>=25
86323	2	1	24	1	1	2	27.7	13.4	19.6055	8	2.483	1.37	4.45	1.30	5.83	<60	94	<25
86334	1	5	72	22	1	1	42.1	14.1	20.9555	9	3.207	1.27	5.38	1.99	17.83	70-80	112	<25
86350	2	1	49	10	1	2	33.7	13.3	20.7055	7.5	3.026	1.45	5.12	1.39	11.83	<60	118	<25
86384	2	3	37	7	1	2	34.3	13.8	35.05	6	3.698	1.06	5.90	2.51	12.61	<60	114	<25
86404	1	3	80	40	1	1	26.8	12.4	18.7055	8	1.293	0.85	2.40	0.59	9.00	>=80	154	>=25
86451	1	2	63	16	1	2	28.2	14.6	22.2055	11	1.345	0.85	3.62	3.11	13.89	60-70	106	<25
86480	1	2	65	4	1	2	25.3	13.4	20.7055	6.7	3.414	1.47	5.48	1.28	9.78	60-70	158	<25
86495	2	5	68	10	1	1	27.5	12.9	18.9055	6.6	1.577	1.03	2.97	0.77	6.78	60-70	142	<25
86674	1	3	62	7	1	2	45	16.5	24.1055	7.4	2.534	1.14	4.55	1.91	10.06	60-70	122	<25
86717	2	3	62	22	1	1	43.3	14.5	21.892	6.1	4.939	1.5	7.11	1.49	9.50	60-70	126	<25
86753	2	3	80	30	1	2	33.2	13.9	21.6895	6.9	2.87	1.34	4.91	1.53	9.72	>=80	124	>=25
86966	2	3	80	20	1	2	31.2	14.7	22.811	7.6	1.681	1.58	4.09	1.83	15.06	>=80	140	<25
87021	1	3	58	17	1	1	41.1	15.7	23.614	7.5	1.862	0.85	3.72	2.21	10.78	<60	108	<25
87078	2	2	63	18	1	2	36	13.1	19.4055	7.1	3.026	1.14	5.07	1.99	7.67	60-70	106	<25
87109	2	5	67	10	1	1	22.6	13.1	19.9555	7.5	1.552	1.42	3.78	1.74	7.89	60-70	122	<25
87132	1	1	39	6	1	2	28.1	16.3	60.4	7	3.336	1.01	6.39	4.46	15.72	<60	118	<25

87155	2	1	71	31	1	2	32.2	14.2	22.0555	7.6	2.276	1.94	4.53	0.70	8.33	70-80	118	>=25
87257	2	5	59	7	1	2	22.9	12.8	19.6635	8	3.595	1.42	5.64	1.36	10.11	<60	176	<25
87284	1	5	80	30	1	2	23.6	12.7	19.363	6.4	1.267	1.6	3.23	0.77	6.95	>=80	112	>=25
87417	1	4	60	22	1	2	36.4	12.6	30.75	10	3	1.09	4.40	0.70	10.95	60-70	134	<25
87706	2	3	80	20	1	2	25.1	12.3	19.4785	6	2.121	1.47	4.27	1.46	5.50	>=80	154	<25
87727	2	2	80	38	1	1	33.3	13.6	20.9555	7	2.922	0.98	4.78	1.90	7.00	>=80	110	>=25
87770	1	3	68	8	1	2	27.5	15.4	22.9765	6.7	2.302	1.11	4.06	1.41	7.06	60-70	146	<25
87824	2	1	54	15	1	1	49.5	11	17.3555	8	2.146	1.32	4.03	1.23	6.06	<60	134	<25
87962	1	5	61	1	1	1	30.9	14.6	22.0555	5.9	1.448	1.24	3.08	0.84	5.83	60-70	120	<25
88011	2	3	64	9	1	2	39.3	14.1	20.9055	6.4	2.483	1.22	4.40	1.53	7.11	60-70	116	<25
88019	1	3	73	13	1	1	31.4	15.3	22.9125	7.6	1.5	0.85	2.51	0.33	13.61	70-80	108	<25
88164	2	2	45	1	1	1	39.3	13	64.8	5.7	3.026	0.96	5.22	2.69	6.39	<60	122	<25
88191	1	2	68	30	1	1	31.4	14.8	21.3055	9	1.577	0.8	3.03	1.40	17.67	60-70	138	>=25
88303	1	5	65	20	1	2	33.2	14.4	30.95	8.1	1.888	1.34	3.88	1.42	8.56	60-70	168	<25
88334	2	4	39	15	1	2	42.4	9.1	14.499	7.4	3.879	0.85	5.79	2.33	10.00	<60	136	<25
88350	1	4	45	10	1	1	33.1	14.6	21.7055	8	3.31	1.94	5.51	0.54	17.28	<60	156	<25
88402	2	4	47	18	1	2	27.7	13.8	20.3555	7.2	2.974	1.47	5.17	1.59	6.72	<60	150	<25
88483	2	5	78	18	1	2	24.4	13.3	20.8055	9	1.81	1.6	3.70	0.64	5.00	70-80	118	<25
88544	1	3	67	5	1	1	40.2	15.4	22.816	10	3.905	0.96	6.80	4.23	17.39	60-70	148	<25
88660	1	3	54	12	1	2	27	17.9	69.5	7.1	1.733	1.03	3.21	0.97	8.33	<60	128	<25
88692	1	3	71	13	1	2	33.9	14.8	21.7285	7.5	2.276	1.06	3.96	1.34	8.28	70-80	104	<25
88719	1	1	61	4	1	2	33	16	23.7555	7	1.836	0.8	3.26	1.33	8.89	60-70	120	<25
88738	2	4	58	3	1	2	36.9	11.7	18.858	7	2.327	1.55	4.19	0.66	5.89	<60	126	<25
88743	1	5	65	2	1	2	23.9	14.5	23.0055	6.1	1.345	1.45	3.44	1.44	6.00	60-70	114	<25
88758	2	4	63	3	1	2	37.2	11.9	18.3555	5.5	2.689	2.25	5.28	0.73	4.78	60-70	122	<25
88859	1	3	50	15	1	2	33.2	13.8	20.1245	8.9	3.776	1.76	6.85	2.89	15.78	<60	170	<25
88874	1	3	64	20	1	2	36	15.2	22.3055	11.1	2.069	0.88	4.01	2.29	11.78	60-70	130	<25
88902	1	3	68	13	1	2	29.4	15.9	42.6	7.8	0.802	1.89	2.97	0.63	12.33	60-70	114	<25
89069	2	5	63	15	1	2	35.4	13.8	20.3555	6.2	2.172	1.34	3.78	0.55	6.17	60-70	128	<25
89090	2	3	56	15	1	2	30.6	12.8	77.1	7	2.327	1.06	4.50	2.45	4.67	<60	124	<25
89158	1	3	55	14	1	2	22.2	14.7	76.95	9.1	2.224	1.91	4.40	0.57	9.89	<60	118	<25
89190	1	4	47	15	1	1	27.9	9.1	14.856	5.4	1.629	1.66	3.65	0.78	7.00	<60	114	<25
89275	2	2	67	25	1	2	57.2	12.9	20.0055	7	1.396	1.14	3.03	1.10	13.17	60-70	168	>=25
89321	2	4	67	4	1	2	28.2	10.6	16.9055	6	2.896	1.27	4.50	0.73	5.67	60-70	102	<25
89422	1	3	80	20	1	2	31.3	13.1	19.4555	6.5	2.146	1.09	4.16	2.05	11.06	>=80	138	<25
89437	1	3	70	6	1	2	29	13.2	20.4055	6.7	1.138	0.7	2.79	2.09	8.39	70-80	156	<25
89565	2	3	55	5	1	2	29.1	14.4	125.8	7	3.465	1.06	5.74	2.67	8.67	<60	122	<25
89639	2	3	29	4	1	2	35.3	13.8	20.758	8.7	2.664	1.03	4.27	1.25	10.67	<60	114	<25
89650	1	4	62	12	1	2	36.4	13	20.5055	8.1	3.284	1.6	5.40	1.14	8.22	60-70	118	<25
89712	2	4	48	10	1	2	34.4	12.4	19.3055	9.8	4.163	1.01	5.53	0.81	5.83	<60	146	<25
89770	2	3	67	6	1	2	37.1	13.5	20.073	7.5	2.819	1.03	4.91	2.33	11.11	60-70	120	<25
89813	2	3	40	5	1	2	32.3	11.6	18.2995	6.9	4.396	0.93	6.31	2.12	6.28	<60	122	<25
89848	1	5	80	11	1	2	23.3	13.9	20.958	6.8	2.198	0.98	3.90	1.59	7.39	>=80	124	<25
89851	1	3	44	17	1	1	43.7	14.3	21.6055	7	3.207	1.06	4.91	1.44	9.61	<60	146	<25
90021	1	5	64	16	1	1	24	14.6	21.6555	11	1.577	0.85	3.41	2.17	8.83	60-70	116	<25
90066	2	5	63	8	1	2	26.7	11.8	111.85	6.2	1.448	1.22	3.52	1.84	8.95	60-70	116	<25
90115	1	3	79	9	1	2	27.1	14.6	22.0605	6.2	1.888	0.98	3.54	1.45	8.83	70-80	118	<25
90256	1	2	51	7	1	1	28.9	15.2	22.8055	18.5	4.008	1.78	6.13	0.72	17.33	<60	144	<25
90257	2	5	69	6	1	2	22.3	13.3	20.1555	7.2	1.577	1.97	3.85	0.70	5.28	60-70	132	<25
90282	2	1	49	3	1	2	31.6	12.4	19.7555	7	4.448	0.8	6.13	1.94	11.61	<60	126	<25
90303	1	3	56	15	1	2	50.6	13.5	20.2055	7.1	2.689	1.4	4.53	0.98	8.56	<60	104	<25
90436	1	4	59	10	1	2	19.2	9.8	14.8085	6.7	3.646	1.84	6.10	1.33	4.00	<60	136	<25
90565	2	3	67	11	1	2	29.4	13.6	20.8555	7.1	1.396	0.91	3.00	1.55	9.39	60-70	140	<25
90700	2	4	40	27	1	2	35.2	12.4	19.6055	9	3.595	1.55	5.56	0.93	11.78	<60	150	>=25
90745	2	3	32	2	1	2	46.2	14.5	21.6055	6.5	2.327	1.22	4.47	2.02	8.50	<60	152	<25
90748	2	1	61	11	1	2	32.5	14.3	20.8055	7.8	2.767	0.88	4.73	2.37	10.33	60-70	126	<25
90783	1	4	47	2	1	1	43.7	15.5	143.6	5.3	2.612	1.24	4.19	0.73	6.50	<60	156	<25
90859	2	2	50	14	1	2	50.3	12.9	19.9055	13	1.5	0.78	2.95	1.49	12.61	<60	106	<25
90922	1	4	59	13	1	2	46.9	15.2	22.6055	6.5	3.465	1.53	5.35	0.78	12.22	<60	104	<25
90938	1	3	55	3	1	2	35.7	15.9	57	9	2.948	1.03	4.84	1.89	11.17	<60	120	<25
91050	2	2	75	20	1	2	36.6	12.2	17.934	8	0.802	0.93	2.17	0.98	19.11	70-80	108	<25
91065	2	2	52	10	1	2	36.8	14.5	21.659	9	5.741	1.03	7.76	2.14	8.89	<60	132	<25
91114	1	1	61	23	1	2	35.1	17.4	25.2055	8	1.862	0.59	4.03	3.42	11.22	60-70	134	<25
91171	2	5	80	15	1	2	23.6	12.8	20.0055	6	1.5	1.5	4.03	2.24	6.00	>=80	130	<25
91187	1	3	63	15	1	2	35.3	14.4	20.3555	8.1	2.819	1.03	4.32	1.01	4.50	60-70	122	<25
91220	1	5	65	32	1	2	23.9	15	22.5555	9.5	1.655	1.14	3.13	0.73	10.00	60-70	94	>=25
91495	1	2	55	18	1	2	38.8	16.1	23.843	6.1	1.707	1.19	3.44	1.19	9.67	<60	116	<25
91771	2	1	39	16	1	2	29.6	11.9	18.7055	7.2	2.845	1.11	4.58	1.38	8.56	<60	110	<25
91846	2	4	56	5	1	2	30.6	12.4	18.5555	6.2	2.431	0.96	4.68	2.85	9.89	<60	176	<25
91899	2	5	48	17	1	2	21	11.7	18.2555	5.5	2.198	2.2	4.58	0.41	4.78	<60	118	<25
91912	1	2	41	1	1	1	55.4	14.5	43.85	13	3.491	0.75	5.09	1.85	6.95	<60	150	<25
91950	1	3	61	21	1	2	27.7	14.2	21.3055	6	1.888	1.16	3.41	0.78	5.72	60-70	104	<25
91970	1	5	59	6	1	2	25.5	14.5	21.7055	7.1	3.129	1.32	5.28	1.79	5.56	<60	116	<25
92048	1	1	75	20	1	2	28.8	17	25.7555	7.7	2.353	1.06	3.90	1.05	5.28	70-80	156	<25
92121	1	4	57	12	1	2	33.5	14.1	22.0055	6.4	1.577	1.32	3.41	1.15	8.28	<60	134	<25
92356	1	4	54	14	1	2	33	15.4	23.1555	7	2.483	0.78	3.54	0.64	8.22	<60	146	<25
92386	2	4	57	8	1	2	32.4	14.2	22.2055	7	4.655	1.94	7.06	1.02	12.22	<60	192	<25
92435	1	2	65	6	1	2	36.7	14	20.5555	5.2	1.914	1.11	3.67	1.41	9.39	60-70	140	<25
92437	1	3	50	2	1	2	31.4	15.9	23.8055	6	1.629	1.14	3.72	2.08	15.83	<60	130	<25
92487	1	3	69	18	1	2	25.7	15.7	23.9055	6.9	2.896	1.14	4.50	1.01	9.83	60-70	144	<25
92500	2	5	56	18	1	2	32.1	14.9	22.313	7.1	2.664	1.37	4.68	1.42	5.56	<60	118	<25
92676	1	4	42	3	1	2	30.2	16.5	25.009	6.5	6.879	1.63	9.28	1.67	25.22	<60	122	<25
92689	1	3	44	9	1	1	31.8	15.9	195.1	8.4	1.888	1.09	3.26	0.63	13.56	<60	114	<25
92696	1	4	55	8	1	2	41.2	11.7	18.4055	7	2.172	1.14	3.80	1.05	5.50	<60	132	<25
92883	1	3	63	3	1	2	32.8	13	19.4055	6.5	1.965	1.27	4.37	2.50	7.83	60-70	120	<25
92988	2	4	51	11	1	2	64.5	12.3	19.8755	6.2	4.06	1.58	5.95	0.67	8.28	<60	136	<25
92989	1	2	65	12	1	1	41.5	13.3	20.4555	7	2.483	1.32	4.60	1.76	9.83	60-70	152	<25
93005	1	3	79	4	1	2	28.1	18	27.6055	7	1.94	1.29	3.52	0.60	8.22	70-80	104	<25
93024	2	4	40	4	1	2	28.2	13.8	21.7555	8.5	3.388	1.37	5.09	0.76	13.89	<60	128	<25
93238	2	1	69	5	1	2	37.2	13.5	21.5055	6.4	1.448	1.16	3.28	1.46	8.17	60-70	114	<25
93258	2	3	80	40	1	2	36.3	9.3	14.3515	6.7	2.121	0.83	3.41	1.02	7.17	>=80	128	>=25
93283	2	4	76	3	1	2	48.2	15	22.9055	7	4.396	1.01	6.28	1.91	6.95	70-80	158	<25
93354	2	2	64	15	1	2	17.7	14.8	102.1	6.7	2.767	2.3	5.51	0.98	8.28	60-70	134	<25
93511	2	4	40	1	1	2	35.3	14.5	22.21	6.5	2.948	1.11	4.60	1.21	16.78	<60	147	<25
93573	2	4	55	22	1	2	37.3	13.6	20.5555	8.1	2.845	1.78	5.09	1.03	6.50	<60	110	<25
93607	1	2	50	5	1	2	33.6	15.3	26.6	6.4	3.103	0.85	5.17	2.67	10.89	<60	140	<25
93656	2	1	75	20	1	2	37.9	14.3	20.9555	7	2.121	1.19	3.93	1.34	7.83	70-80	136	<25
93689	2	1	69	2	1	2	28.1	13.1	19.6555	7	1.888	2.09	4.32	0.72	6.00	60-70	164	<25
